# Medication adherence trajectories and clinical outcomes in patients with cardiovascular disease: a systematic review and meta-analysis

**DOI:** 10.7189/jogh.15.04145

**Published:** 2025-05-09

**Authors:** Qiaoling Hou, Yuhan Zhao, Ying Wu

**Affiliations:** 1 School of Nursing, Capital Medical University, Beijing, China

## Abstract

**Background:**

The role of medication adherence trajectories in the clinical outcomes in patients with cardiovascular diseases (CVDs) remains unclear. We aimed to analyse the impact of different medication adherence trajectories on mortality and other key clinical outcomes in patients with CVDs.

**Methods:**

We identified longitudinal cohort studies that reported the association between medication adherence trajectories and clinical outcomes in patients with CVDs by conducting a comprehensive search of the Cochrane Library, PubMed, Embase, CINAHL, and Web of Science databases, without applying language restrictions in August 2024. We pooled the published hazard ratios and 95% confidence intervals using random effects models and assessed potential bias through Egger regression analysis.

**Results:**

In this meta-analysis, we included nine cohorts with 226 203 patients with a mean age of 66.1 years and a maximum follow-up of five years. Eight of the nine studies used the proportion of days covered to assess medication adherence. We identified four distinct medication adherence trajectories: persistent adherence, persistent nonadherence, gradually increasing adherence, and gradually declining adherence. Compared to persistent adherence, persistent nonadherence was associated with significantly higher risks of all-cause mortality, major adverse cardiovascular events (MACE), and recurrent venous thromboembolism, with risk increases ranging from 32% to nearly three times higher. Gradually increasing adherence was associated with a 26% higher risk of mortality and a 22% increased risk of MACE. In the group with gradually declining adherence, the risk of MACE increased by 24%, while the risk of major bleeding decreased by 43%. The overall risk of bias was low. Sensitivity analyses confirmed the consistency and robustness of these findings.

**Conclusions:**

This study underscores the substantial benefits of maintaining persistent adherence to prescribed medication regimens for patients with CVDs. Conversely, persistent nonadherence significantly elevates the risk of adverse clinical outcomes.

**Registration:**

PROSPERO: CRD42023456395.

Medication adherence plays a central role in the effective management of cardiovascular diseases (CVDs), the leading cause of death globally, accounting for an estimated 17.9 million deaths annually, or 32% of all global mortality [1,2]. Evidence has shown that improved adherence to prescribed medications significantly mitigates the risk of adverse cardiovascular events, reduces health care costs, improves overall health outcomes, and reduces mortality by 35% [3]. However, non-adherence continues to pose a significant challenge, with approximately 30% of prescriptions unfilled and 50% of initiated treatments discontinued within the first year [4,5]. This widespread non-adherence exacerbates the burden of CVDs, contributing to increased rates of hospitalisations, emergency department visits, and mortality while placing substantial economic pressure on health care systems [6].

Although many studies have been conducted to examine the nature of medication adherence, our understanding of non-adherence remains limited. Traditional research has primarily focussed on static assessments, such as adherence levels at a single time or averaged over fixed periods [7–10], failing to capture the dynamic nature of medication adherence, which fluctuates throughout the treatment course. Measures like the proportion of days covered are limited in accounting for evolving adherence patterns during initiation, maintenance, and potential discontinuation phases [11].

To address these limitations, group-based trajectory modelling has emerged as an innovative method for evaluating medication adherence over time. Group-based trajectory modelling identifies distinct adherence trajectories within a patient population, offering a nuanced understanding of how adherence patterns evolve and relate to clinical outcomes. Unlike static measures, group-based trajectory modelling captures the complexities of adherence behaviour, including periods of non-adherence or gradual changes, which are critical for understanding long-term treatment success [12–14].

Several meta-analyses have evaluated the impact of medication adherence trajectories in chronic diseases, including CVD. However, these reviews have notable limitations. First, most reviews focus on adherence trajectories and their predictors but fail to explore how these trajectories influence clinical outcomes [6,7,15]. Second, the specific relationship between adherence trajectories and clinical outcomes remains unclear. Some studies suggest that a gradual increase in adherence, compared to persistent adherence, is linked to a higher incidence of major adverse cardiovascular events (MACE) and poorer clinical outcomes [16,17]. Others report elevated MACE risk in patients whose adherence gradually declines or increases [18,19].

Given these gaps in the literature, we aimed to conduct a systematic review and meta-analysis to evaluate the impact of longitudinal medication adherence trajectories on clinical outcomes in CVD patients. By examining how adherence patterns evolve over time and their influence on health outcomes, we aimed to provide valuable insights into the adherence-clinical outcome relationship. These findings will enhance our understanding and support the development of more personalised, dynamic strategies for managing CVD effectively.

## METHODS

### Study design

We registered the study protocol on PROSPERO (CRD42023456395), and followed the Cochrane Handbook of Systematic Reviews of Intervention and the PRISMA guidelines [20,21].

### Search strategy

We conducted a thorough literature search to identify prospective and retrospective cohort studies related to medication adherence trajectories and clinical outcomes in patients with CVD. We searched studies published in the Cochrane Library, PubMed, Embase, CINAHL, and Web of Science databases from their inception to 15 August 2024 without language restrictions (Section 1 in the **Online Supplementary Document**). In addition, we manually reviewed the reference lists of the included full texts, performed a supplementary search on the ClinicalTrials.gov trial registration platform, and contacted the authors for further information if needed.

We constructed search terms by combining subject words with free words. The key terms included ‘Coronary Disease’, ‘Coronary Artery Disease’, ‘Myocardial Ischemia’, ‘Acute Coronary Syndrome’, ‘Coronary Artery Bypass’, ‘Myocardial Revascularization’, ‘Percutaneous Coronary Intervention’, and ‘Pharmaceutical Preparations’, ‘Polypharmacy’, ‘Prescription Drugs’, and ‘Patient Compliance’, ‘Medication Adherence’.

### Selection criteria

We selectively included studies that met the following criteria: they must employ a longitudinal observational design; they must be conducted in adult patients with CVD, aged ≥18 years; they should report on the associations between at least two distinct medication adherence trajectories and clinical outcomes; and the results must be presented using any quantifiable measure of relative risk. We excluded studies with incomplete information, systematic reviews, meta-analyses, and reports from meetings or congresses.

### Study selection

All studies retrieved from the database search were managed and organised by exporting the citations to EndNote X9 (Clarivate, Philadelphia, Pennsylvania, USA) and Rayyan (Rayyan Systems Inc., Cambridge, Massachusetts, USA) [22]. Microsoft Excel was used for data extraction. Two reviewers (QLH and YHZ) independently screened the titles, abstracts, and full study texts. Disagreements between the reviewers regarding the title and abstract screening, full-text review, and reasons for exclusion were resolved by discussion with the third reviewer (YW).

### Data extraction

Two reviewers (QLH and YHZ) independently extracted data on the characteristics of the study cohort study information (*i.e.* author, year, country of origin, sample size, cohort type, CVD type, mean age), primary and secondary outcomes (definition of medication adherence trajectories, years of follow up, reverse causation analysis), and key information for the risk-of-bias assessment. The data extraction table was pilot tested by QLH with three studies (reviewed by YHZ). This pilot testing process aimed to assess the reliability and clarity of the extraction form. Feedback from this step identified areas for improvement, such as clarifying certain data fields and refining instructions for specific variables. Based on these findings, the extraction protocol was revised to enhance consistency and accuracy. Next, authors QLH and YHZ retrieved data from the full-text studies, and conflicts were resolved by discussion and consultation with the third author (YW).

### Risk of bias assessment

To determine the risk of bias, two reviewers (QLH and YHZ) independently applied the Newcastle-Ottawa Scale (NOS) to the selected studies (Section 2 in the **Online Supplementary Document**) [23]. A total score is calculated by summing up the star ratings assigned to each item, with higher scores indicating a lower risk of bias. Specifically, we considered studies that scored eight or nine on the NOS to have a low risk of bias, and those scoring 4–7 to have a moderate risk of bias. Studies scoring 0–3 are classified as having a high risk of bias [24].

### Data synthesis and statistical analysis

We conducted a narrative synthesis of the findings from the included studies. We used published aggregated data from each study and applied the inverse variance weighted method to combine [25] the extracted hazard ratios (HRs) and their 95% confidence intervals (CIs) using random-effects models to account for the effect of between-study heterogeneity [26]. Heterogeneity was evaluated using the *I*^2^ statistic and defined as 0–40%: might not be important; 30–60%: may represent moderate heterogeneity; 50–90%: may represent substantial heterogeneity; 75–100%: considerable heterogeneity [27]. We evaluated the publication bias and small study effects using funnel plots and Egger regression symmetry tests [28]. Statistical analyses were performed in Stata MP, version 17.0 (StataCorp LLC, College Station, Texas, USA).

We performed subgroup analyses for different mean ages (<65 years and ≥65 years) to investigate the heterogeneity across several subgroups.

### Sensitivity analyses

We conducted several sensitivity analyses to ensure the robustness of our findings. To evaluate the influence of individual studies on our overall results, we systematically excluded each study one at a time and recalculated the pooled risk estimates and heterogeneity. This ‘leave-one-out analysis’ helped identify any single study that might disproportionately affect our findings.

We performed random-effects meta-regression analyses to explore sources of between-study heterogeneity. We considered variables such as sex, follow-up duration, disease type, medication, and age groups independent variables, with risk estimates as the dependent variable. Specifically, we conducted a stratified analysis based on the mean age of the cohorts, using 65 years as the cutoff. This age threshold is commonly used in cardiovascular research to distinguish between younger and older populations, as the incidence and outcomes of CVDs can vary significantly across these age groups.

To account for temporal trends, we conducted cumulative meta-analyses for each outcome, stratified by the year of publication and follow-up duration. This allowed us to observe how the accumulation of evidence over time influenced the overall effect estimates. Recognising that patients might discontinue medications due to adverse reactions, potentially leading to unfavourable clinical outcomes, we performed a sensitivity analysis limited to studies that accounted for reverse causation. This approach aimed to isolate the impact of medication adherence trajectories on clinical outcomes, minimising the confounding effect of discontinuation due to adverse effects.

Finally, to assess the potential influence of study quality on our results, we restricted analyses to studies with a low risk of bias. This ensured that our findings were not unduly affected by the included studies' methodological limitations.

These sensitivity analyses were integral in confirming the robustness and reliability of our meta-analysis findings.

## RESULTS

### Identification of relevant studies

The search and screening process yielded substantial literature for potential inclusion. After removing duplicates, we identified 14 464 citations. Initial screening of titles and abstracts narrowed the selection to 42 studies for full-text evaluation. Of these, 33 were excluded as they did not analyse the trajectories of medication adherence (Figure S1 in the **Online Supplementary Document**). Ultimately, nine studies met all inclusion criteria and were incorporated into our systematic review and meta-analysis. Of these, three were prospective studies [16,18,19], and six were retrospective [17,29–33], with findings reported as HRs.

### General characteristics of the included studies

We included nine studies, encompassing 226 203 patients (**Table 1**). Sample sizes varied from 6597 to 101 011 across the studies. The weighted average age of participants was 66.1 years. The percentage of female participants ranged from 23.6% to 58.8% in studies that included both sexes. There were three prospective cohort studies and six retrospective cohort studies. Two studies examined the impact of multi-drug adherence on clinical outcomes, while other studies focussed on adherence to specific drugs: two on statins, one on antiplatelet therapy, two on warfarin, and two on oral anticoagulants other than warfarin. The follow-up periods across studies varied from six months to five years. Regarding quality assessment, the nine non-randomised studies achieved scores between seven and nine out of nine on the Newcastle-Ottawa Scale, indicating high methodological quality.

**Table 1:** General characteristics of the included studies

Study, year, reference	Country	First year of recruitment	Number of participants	Cohort type	CVD type	Female, %	Age, x̄	Comorbidities, %	Adjustment for
Turgeon et al., 2022 [16]	Canada	2012	12 844	Prospective	ACS	23.6	62.4	Diabetes = 25, hypertension = 65.5	Age, sex, socioeconomic status, ACS subtype, comorbidities
Rodríguez-Bernal et al., 2022 [17]	Spain	2009	15 797	Retrospective	ACS (AMI and angina)	28	68	Hypertension = 61.57, diabetes = 34.13, lipid disorder = 50.59, CHD = 25.40, COPD = 11.70	Age at hospital admission, gender
May et al., 2022 [18]	USA	1999	7339	Prospective	ASCVD includes CAD, cerebrovascular disease, and PAD	23.5	56.4	Hypertension = 64.9–70.3, hyperlipidaemia = 69.7–9.9, diabetes = 25.8–28.8, smoking = 33.7–40.1, depression = 9.1–13.3	Age, sex, index ASCVD event, comorbidities, socioeconomic status, and area deprivation index
Kumbhani et al., 2013 [19]	Multiple countries	2003	37 154	Prospective	Established atherothrombosis: coronary, cerebrovascular, and PAD	32	68.2	Diabetes mellitus: 37.3 (adherent), 36.4 (nonadherent), hypercholesterolemia: 93.0 (adherent), 46.5 (nonadherent) hypertension: 82.5 (adherent), 76.8 (nonadherent)	Gender, age current smoking status, History of diabetes, body mass index, timing of ischaemic event (≤1 year or >1 year), geographic regions
Hickson et al., 2019 [29]	USA	2008	101 011	Retrospective	AMI	54.3	76.6	CABG = 1.1, ischaemic heart disease = 59.7, unstable angina = 5.3	Age, sex, race/ethnicity, dual eligibility in Medicare and Medicaid, median household income
An et al., 2022 [30]	USA	2012	18 920	Retrospective	AF	42.8	72.6	Diabetes = 27.5, hypertension = 61.2	Age, sex, race, ethnicity, peripheral vascular disease, myocardial infarction
Kang et al., 2023 [31]	USA	2013	10 448	Retrospective	VTE	50.6	59.6	MI = 9.9, AF = 15.0	Age, sex, comorbidities (such as cancer, surgery, trauma, hyperlipidaemia, respiratory diseases)
Kang et al., 2023 [32]	USA	2013	16 093	Retrospective	VTE	48.9	58.7	AF = 13.1, heart failure = 17.8, MI = 9.5	Age, sex, comorbidities (such as cancer, surgery, trauma, hyperlipidaemia, respiratory diseases)
Park et al., 2023 [33]	USA	2014	6597	Retrospective	VTE	58.8	72.6	Hyperlipidaemia = 69.2, ischaemic heart disease = 47.3, MI = 18.8, AF = 24.6	Age, sex, race, hyperlipidaemia, chronic kidney disease, cancer, surgery

ACS – acute coronary syndrome, AF – atrial fibrillation, AMI – acute myocardial infarction, ASCVD – atherosclerotic cardiovascular disease, CABG – coronary artery bypass surgery, CAD – coronary artery disease, CHD – coronary heart disease, COPD – chronic obstructive pulmonary disease, MI – myocardial Infarction, PAD – peripheral artery disease, VTE – venous thromboembolism, x̄ – mean

Among the nine studies included in our analysis, five were conducted in patients with CHD [16–19,29], one focussed on patients with atrial fibrillation [30], and the remaining three examined patients with venous thromboembolism (VTE) [31–33]. These studies rigorously adjusted for potential confounders to ensure robustness in the observed associations. The adjusted variables included sex, follow-up duration, disease type, medication, and age groups.

### Measurement of the exposure

Among the nine studies included, eight used the proportion of days covered metric to assess medication adherence, while one relied on self-reported data. All studies assessed medication adherence trajectories by comparing adherence status at both baseline and follow-up. We categorised adherence into four longitudinal nominal categories: persistent adherence, persistent non-adherence, gradual decline, and gradual increase (Table S1 in the **Online Supplementary Document**). ‘Gradual decline’ was defined as transitioning from the adherent category to the non-adherent category, and ‘gradual increase’ as the reverse transition. Four studies used persistent adherence as the reference category, while the remaining five used persistent non-adherence. The reference category for these five studies was converted to ‘persistent adherence’ by applying the multiplicative inverse of their reported HRs.

### Measurement of the outcomes

Clinical outcomes assessed included all-cause mortality (three studies), MACE (three studies), recurrent VTE (four studies), and major bleeding (five studies).

### Potential bias and quality assessment

Egger tests revealed no significant evidence of publication bias for all-cause mortality (*P* = 0.058), MACE (*P* = 0.207), and recurrent VTE rates (*P* = 0.189) (Figure S2–4 in the **Online Supplementary Document**). However, the test indicated potential publication bias for major bleeding (*P* = 0.015) (Figure S5 in the **Online Supplementary Document**). The initial inter-rater agreement on study quality was 90%, and a full consensus was reached after the discussion. Study quality scores ranged from seven to nine out of nine, with seven studies categorised as low risk of bias and two as moderate risk of bias (Table S2 in the **Online Supplementary Document**). The reduced quality in two studies was due to unclear descriptions of the non-exposed group sourcing, and one study’s reliance on self-reported outcomes impacted its quality assessment in selection and outcome domains.

### Medication adherence trajectories and all-cause mortality

**Figure 1:**
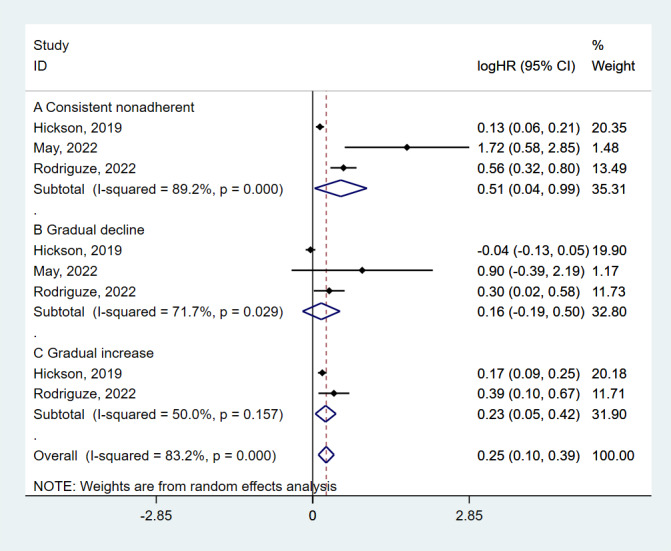
Meta-analysis on the associations of medication adherence trajectories with all-cause mortality.

Three studies on the impact of changes in medication adherence on all-cause mortality found significant associations. Persistently non-adherent patients had a significantly higher risk of all-cause mortality (HR = 1.67; 95% CI = 1.04–2.68, *P* = 0.035) (**Figure 1**). Those with gradually increasing adherence had a 26% higher risk (HR = 1.26; 95% CI = 1.05–1.53, *P* = 0.014).

### Medication adherence trajectories and MACE incidence rate

**Figure 2:**
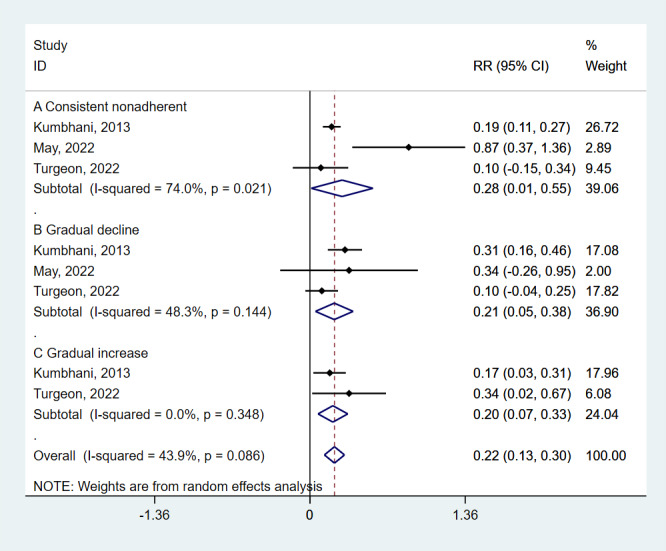
Meta-analysis on the associations of medication adherence trajectories with MACE incidence rate. MACE – major adverse cardiovascular events.

Three studies examined the relationship between changes in medication adherence and MACE incidence. Compared to the persistent adherence group, those who were persistently non-adherent had a 32% higher risk of MACE (HR = 1.32; 95% CI = 1.01–1.73, *P* = 0.041) (**Figure 2**). The gradually declining adherence group had a 24% increased risk (HR = 1.24; 95% CI = 1.05–1.46, *P* = 0.011), and the gradually increasing adherence group had a 22% increased risk (HR = 1.22; 95% CI = 1.07–1.40, *P* = 0.002).

### Medication adherence trajectories and recurrent VTE incidence rate

**Figure 3:**
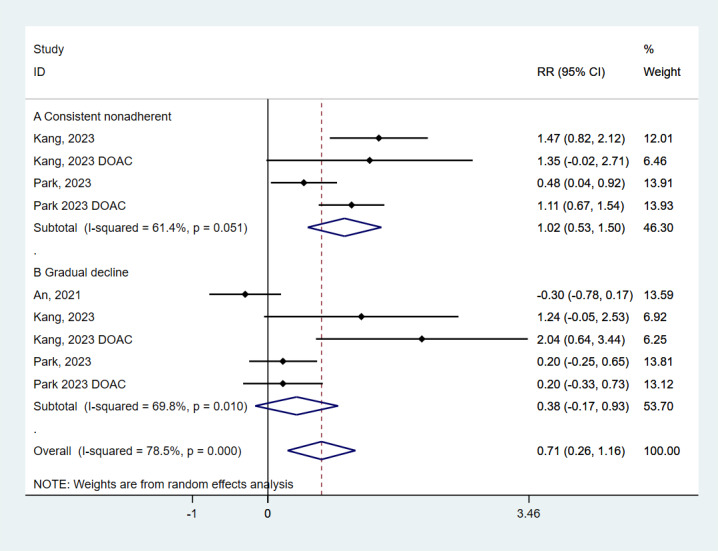
Meta-analysis on the associations of medication adherence trajectories with the risk of recurrent VTE incidence rate. VTE – venous thromboembolism.

Persistently non-adherent patients had a 2.76 times higher risk compared to those with persistent adherence (HR = 2.76; 95% CI = 1.70–4.50, *P* < 0.001) (**Figure 3**). Those with a gradual decline in adherence had a 46% higher risk, though this was not statistically significant (HR = 1.46; 95% CI = 0.84–2.54, *P* = 0.174).

### Medication adherence trajectories and major bleeding incidence rate

Compared to persistent adherent patients, those with persistent non-adherence showed no increased risk (HR = 0.82; 95% CI = 0.54–1.24, *P* = 0.342) (Figure S6 in the **Online Supplementary Document**). Interestingly, patients with gradual adherence had a 43% lower risk of major bleeding (HR = 0.57; 95% CI = 0.35–0.90, *P* = 0.017).

### Sensitivity analysis

Our sensitivity analyses involved several robust checks to validate the consistency and reliability of our findings. Initially, excluding any single study from the meta-analyses did not alter the overall results, indicating robustness across various studies (Figure S7 in the **Online Supplementary Document**). Sex and time of follow-up years were identified as significant sources of heterogeneity for the risk of MACE incidence rate in the meta-regression analysis (Figure S10 in the **Online Supplementary Document**). Further stratified analyses demonstrated consistent results in cohorts ≥65 years, with no significant changes observed in the patterns of outcomes (Figures S8 and S9 in the **Online Supplementary Document**). However, for CVD patients <65 years, the incidence of recurrent VTE was significantly higher in the gradual decline group compared to those with persistent adherence.

Cumulative meta-analyses segmented by the year of publication and duration of follow-up showed no evidence of secular trends affecting clinical outcomes, underscoring the temporal stability of the observed effects (Figures S11–18 in the **Online Supplementary Document**). The robustness of the results was further affirmed after limiting the analyses to studies that accounted for reverse causation and those classified as having a low risk of bias, confirming the reliability of our findings (Figures S19 and S20 in the **Online Supplementary Document**).

## DISCUSSION

### Main findings

This systematic review and meta-analysis included nine longitudinal cohort studies involving 226 203 patients with CVDs, evaluating the relationship between medication adherence trajectories and clinical outcomes. We analysed four adherence trajectories: persistent adherence, persistent non-adherence, gradually declining adherence, and gradually increasing adherence. The findings underscore the critical role of medication adherence in improving clinical outcomes, including reduced risks of all-cause mortality, MACE, and recurrent VTE.

Persistently non-adherent patients exhibited significantly higher risks across multiple outcomes, including a 67% increased risk of all-cause mortality, a 32% elevated risk of MACE, and a nearly 3-fold higher risk of recurrent VTE. These findings translate into substantial clinical consequences, such as higher rates of preventable hospitalisations, worsening cardiovascular conditions, and an increased economic burden due to greater hospital admissions and long-term health care costs. These results highlight the need for robust interventions to improve adherence and mitigate these risks.

Interestingly, patients with gradually declining adherence demonstrated a reduced risk of major bleeding events, potentially attributable to decreased anticoagulant exposure. This finding underscores the nuanced trade-off between the benefits of thrombotic prevention and the risks of bleeding associated with anticoagulation therapy.

### Clinical implications

The observed decline in bleeding risk with reduced anticoagulant adherence is clinically plausible [34,35]. Anticoagulants are effective in preventing thrombotic events but increase bleeding risk. A decline in adherence may result in sub-optimal dosing, which lowers therapeutic efficacy and reduces bleeding risk. This highlights the importance of balancing thrombotic and bleeding risks when managing patients on anticoagulation therapy. Clinicians should adopt patient-centred approaches by considering individual risk profiles, closely monitoring adherence, and adjusting therapy as necessary. For instance, patients with a high bleeding risk may benefit from alternative strategies, such as direct oral anticoagulants with favourable safety profiles or dose adjustments.

To further improve outcomes, proactive management tools, such as digital adherence platforms or routine monitoring during follow-ups, can identify adherence declines early, enabling timely intervention. Early detection of adherence patterns can help prevent adverse events, optimise therapy, and enhance the safety-efficacy balance of treatments like anticoagulation therapy.

### Value of trajectory-based analysis

This study emphasises the unique value of trajectory-based analysis over static adherence measures. Longitudinal analysis provides deeper insights into how adherence evolves over time and its impact on clinical outcomes. By identifying patients at risk of non-adherence or experiencing a decline in adherence, health care providers can more effectively target interventions at critical stages of treatment. These interventions could include personalised counselling, medication adjustments, or the implementation of digital tools like smartphone apps or electronic pill dispensers.

Future research should explore how early identification of adherence trajectories can guide interventions and optimise timing to prevent adverse outcomes. Understanding when and how to intervene can enhance medication adherence and improve long-term outcomes for CVD patients.

### Study strengths

This is the first meta-analysis to comprehensively evaluate the longitudinal associations between medication adherence trajectories and clinical outcomes in CVD patients. The included studies used multiple adherence assessments over time, providing robust data on adherence patterns. We applied stringent criteria to ensure the quality of included studies, which were assessed to have a low to moderate risk of bias. Additionally, sensitivity analyses consistently reinforced our findings, improving confidence in the robustness of the results.

### Study limitations

This study has several limitations. First, heterogeneity among the included studies, such as variations in adherence measurement methods and follow-up duration, may introduce bias. The lack of standardised adherence assessment tools limits comparability and generalisability.

Second, the observational design of the included studies precludes definitive conclusions about causality between adherence trajectories and clinical outcomes. Future randomised controlled trials are needed to strengthen causal inferences.

Third, the few studies included in this meta-analysis (n = 9) limited statistical power, particularly for subgroup analyses. Therefore, we did not perform subgroup analyses according to cohorts, disease subtypes, and medication types.

Fourth, six of the nine studies were conducted in the USA, potentially limiting the generalisability of our findings to other regions with different health care systems and cultural contexts. This geographic concentration underscores the need for research in low- and middle-income countries to understand the impact of adherence trajectories globally.

### Future directions

To optimise medication adherence trajectories and their impact on CVD outcomes, future research should prioritise the following:

− Standardisation of adherence measurements: Using reliable tools like the proportion of days covered or medication possession ratio across studies will improve comparability and reduce heterogeneity, facilitating meta-analyses with more robust conclusions.
− Global research representation: Future studies should include larger and more diverse populations, especially from low- and middle-income countries, to improve the global applicability of findings. Prospective cohort studies or randomised trials with longer follow-up periods are also needed to better establish causal relationships between adherence trajectories and clinical outcomes.
− Routine monitoring and adherence support programmes: Healthcare systems should implement routine adherence monitoring and support programmes, including digital tools and regular follow-ups, to help maintain persistent adherence and improve patient outcomes.
− Innovative monitoring strategies: Emerging technologies like smartphone apps, wearable devices, and electronic pill dispensers offer valuable opportunities for real-time adherence monitoring. Future research should focus on using these tools to track adherence, detect deviations early, and enable timely interventions. Integrating them into clinical practice could shift CVD management from reactive to proactive care, improving long-term outcomes.

## CONCLUSIONS

This meta-analysis reinforces the critical importance of persistent medication adherence in managing cardiovascular diseases. Patients with persistent adherence have significantly better clinical outcomes, including lower risks of mortality, MACE, and recurrent VTE. Conversely, persistent non-adherence is associated with significantly worse outcomes. This study provides a dynamic understanding of adherence trajectories, highlighting the importance of early identification and targeted interventions to improve adherence. Future research should focus on developing strategies to sustain adherence and reduce the global burden of CVDs.

## Additional material


Online Supplementary Document
