# Supplementary material for: Medication adherence trajectories and clinical outcomes in patients with cardiovascular disease: a systematic review and meta-analysis
Source: J Glob Health. 2025 May 9;15:04145. doi: 10.7189/jogh.15.04145 (PMC12061002; doi:10.7189/jogh.15.04145)
Supplement: Online Supplementary Document [file jogh-15-04145-s001.pdf]

Supplement to: Hou Q, Zhao Y, Wu Y. Medication adherence trajectories and clinicaloutcomes in patients with cardiovascular disease: a systematic review and meta-analysis. J Glob Health. 2025;15:04145.

Table of contents

Supplementary section 1: methods 4

a. Search algorithms. 4

Literature search in PubMed 4

Literature search in Cochrane 2

Literature search in Embase 4

b. Figure S1. PRISMA 2020 Flow Diagram for new systematic reviews 6

c. Table S1. Measurement and definition of exposures of included studies reporting on the association of medication adherence trajectories with clinical outcomes in patients with CVD. 6

Supplementary section 2: Assessment of quality and risk of bias results 12

a. Figure S2. Funnel plots for all-cause mortality 12

b. Figure S3. Funnel plots for MACE incidence rate 12

c. Figure S4. Funnel plots for Recurrent Venous Thromboembolism 13

d. Figure S5. Funnel plots for major bleeding 13

e. Egger test for small-study effects in studies reporting all-cause mortality 13

f. Egger test for small-study effects in studies reporting MACE incidence rate 14

g. Egger test for small-study effects in studies reporting Recurrent Venous Thromboembolism 14

h. Egger test for small-study effects in studies reporting Major bleeding 14

i. Table S2. Assessment of quality and risk of bias according to the Newcastle-Ottawa scale. 15

Supplementary section 3: Medication adherence trajectories and major bleeding incidence rate 16

Figure S6. Meta-analysis on the associations of medication adherence trajectories with the risk of major bleeding incidence rate. 16

Supplementary section 4: Sensitivity analyses 16

Figure S7. Sensitivity analysis given named study is omitted. 16

a. consistent nonadherent. All-cause mortality 16

b. gradual decline. All-cause mortality 16

d. consistent nonadherent. MACE incidence rate 17

e. gradual decline. MACE incidence rate 18

f. gradual increase. MACE incidence rate 18

g. consistent nonadherent. Recurrent Venous Thromboembolism 19

h. gradual decline. Recurrent Venous Thromboembolism 19

i. consistent nonadherent. Major bleeding 20

j. gradual decline. Major bleeding 20

Figure S8. Random-effects meta-analysis of Recurrent Venous Thromboembolism according to age. 21

a. Random-effects meta-analysis of Recurrent Venous Thromboembolism in cohorts with mean age <65 years. 21

b. Random-effects meta-analysis of Recurrent Venous Thromboembolism in cohorts with mean age ≥65 years. 22

Figure S9. Random-effects meta-analysis of major bleeding according to age. 22

a. Random-effects meta-analysis of major bleeding in cohorts with mean age <65 years. 22

b. Random-effects meta-analysis of major bleeding in cohorts with mean age ≥65 years. 23

Figure S10. Meta-regressions to explore potential sources of heterogeneity. 23

a. all-cause mortality 23

b. MACE 23

c. recurrent VTE 24

d. Major bleeding 24

Figure S11. Cumulative random-effects meta-analysis of all-cause mortality according to year of publication. 25

a. Consistent nonadherent 25

b. Gradual decline 25

c. Gradual increase 25

Figure S12. Cumulative random-effects meta-analysis of MACE incidence rate according to year of publication. 26

a. Consistent nonadherent 26

b. Gradual decline 26

c. Gradual increase 26

Figure S13. Cumulative random-effects meta-analysis of Recurrent Venous Thromboembolism incidence rate according to year of publication. 27

a. Consistent nonadherent 27

b. Gradual decline 27

Figure S14. Cumulative random-effects meta-analysis of major bleeding incidence rate according to year of publication. 27

a. Consistent nonadherent 27

b. Gradual decline 27

Figure S15. Cumulative random-effects meta-analysis of all-cause mortality according to time of follow up. 28

a. Consistent nonadherent 28

b. Gradual decline 28

c. Gradual increase 28

Figure S16. Cumulative random-effects meta-analysis of MACE incidence rate according to time of follow up. 29

a. Consistent nonadherent 29

b. Gradual decline 29

c. Gradual increase 29

Figure S17. Cumulative random-effects meta-analysis of Recurrent Venous Thromboembolism according to time of follow up. 29

a. Consistent nonadherent 29

b. Gradual decline 30

Figure S18. Cumulative random-effects meta-analysis of major bleeding according to time of follow up. 30

a. Consistent nonadherent 30

b. Gradual decline 30

Figure S19. Meta-analyses restricted to studies accounting for reverse causation. 31

a. Recurrent Venous Thromboembolism 31

b. Major bleeding 31

Figure S20. Meta-analyses restricted to studies with low risk of bias. 31

a. all-cause mortality 31

Supplementary section 5. PRISMA 2020 checklist 33

Supplementary section 1: methods

a. Search algorithms.

Literature search in PubMed

No.	Search Details	Results
#1	((((("Coronary Disease"[Mesh] OR "Coronary Artery Disease"[Mesh]) OR "Myocardial Ischemia"[Mesh]) OR "Acute Coronary Syndrome"[Mesh]) OR "Coronary Artery Bypass"[Mesh]) OR "Myocardial Revascularization"[Mesh]) OR "Percutaneous Coronary Intervention"[Mesh]	510,082
#2	(((((((((((((((((((((((((((((((((((((Coronary Diseases[Title/Abstract]) OR (Disease*, Coronary[Title/Abstract])) OR (Coronary Heart Disease*[Title/Abstract])) OR (Disease*, Coronary Heart[Title/Abstract])) OR (Heart Disease*, Coronary[Title/Abstract])) OR (Ischemia*, Myocardial[Title/Abstract])) OR (Myocardial Ischemia*[Title/Abstract])) OR (Ischemic Heart Disease*[Title/Abstract])) OR (Heart Disease*, Ischemic[Title/Abstract])) OR (Disease*, Ischemic Heart[Title/Abstract])) OR (Artery Disease*, Coronary[Title/Abstract])) OR (Coronary Artery Disease*[Title/Abstract])) OR (Left Main Coronary Artery Disease[Title/Abstract])) OR (Left Main Disease*[Title/Abstract])) OR (Left Main Coronary Disease[Title/Abstract])) OR (Coronary Arterioscleros*[Title/Abstract])) OR (Coronary Atheroscleros*[Title/Abstract])) OR (Acute Coronary Syndrome*[Title/Abstract])) OR (Coronary Syndrome*, Acute[Title/Abstract])) OR (Syndrome*, Acute Coronary[Title/Abstract])) OR (Coronary Intervention*, Percutaneous[Title/Abstract])) OR (Intervention*, Percutaneous Coronary[Title/Abstract])) OR (Percutaneous Coronary Interventions[Title/Abstract])) OR (Percutaneous Coronary Revascularization*[Title/Abstract])) OR (Coronary Revascularization*, Percutaneous[Title/Abstract])) OR (Revascularization*, Percutaneous Coronary[Title/Abstract])) OR (Percutaneous Transluminal Coronary Angioplast*[Title/Abstract])) OR (Artery Bypass*,	281,330

	Coronary[Title/Abstract])) OR (Bypass*, Coronary Artery[Title/Abstract])) OR (Coronary Artery Bypasses[Title/Abstract])) OR (Coronary Artery Bypass Surgery[Title/Abstract])) OR (Coronary Artery Bypass Grafting[Title/Abstract])) OR (Aortocoronary Bypass*[Title/Abstract])) OR (Bypass*, Aortocoronary[Title/Abstract])) OR (Bypass Surgery, Coronary Artery[Title/Abstract])) OR (Myocardial Revascularizations[Title/Abstract])) OR (Revascularization*, Myocardial[Title/Abstract])) OR (Internal Mammary Artery Implantation[Title/Abstract])	
#3	#1 OR #2	603,213
#4	"Pharmaceutical Preparations"[MeSH Terms] OR "Polypharmacy"[MeSH Terms] OR "Prescription Drugs"[MeSH Terms]	931,938
#5	"medication*"[All Fields] OR "regimen*"[All Fields] OR "prescription*"[All Fields] OR "prescribed*"[All Fields] OR "drug*"[All Fields] OR "pill*"[All Fields] OR "tablet*"[All Fields]	7,254,180
#6	#4 OR #5	7,542,298
#7	"Patient Compliance"[MeSH Terms] OR "Medication Adherence"[MeSH Terms]	85,757
#8	"adher*"[All Fields] OR "non adher*"[All Fields] OR "nonadher*"[All Fields] OR "complian*"[All Fields] OR "non complian*"[All Fields] OR "noncomplian*"[All Fields] OR "persisten*"[All Fields] OR "compl*"[All Fields] OR "concord*"[All Fields]	8,435,505
#9	#7 OR #8	8,436,387
#10	"Mortality"[MeSH Terms]	423,921
#11	"mortalit*"[All Fields] OR "death*"[All Fields] OR "fatalit*"[All Fields]	2,241,507
#12	#10 OR #11	2,341,964
#13	#3 AND #6 AND #9 AND #12 NOT "Animals"[MeSH Terms]) NOT ("editorial"[Publication Type] OR "letter"[Publication Type] OR "case reports"[Publication Type] OR "comment"[Publication Type] OR "review"[Publication Type])	422

Literature search in Cochrane

No.	Search Details	Results
#1	Mesh descriptor: [Coronary Disease] explode all trees	18,439
#2	Mesh descriptor: [Myocardial Ischemia] explode all trees	37,016
#3	Mesh descriptor: [coronary artery disease] explode all trees	9,439
#4	Mesh descriptor: [Acute Coronary Syndrome] explode all trees	3,294

#5	Mesh descriptor: [Percutaneous Coronary Intervention] explode all trees	8,428
#6	Mesh descriptor: [Coronary Artery Bypass] explode all trees	6,240
#7	Mesh descriptor: [Myocardial Revascularization] explode all trees	10,697
#8	(Coronary Diseases):ti,ab,kw OR (Disease*, Coronary):ti,ab,kw OR (Coronary Heart Disease*):ti,ab,kw OR (Disease*, Coronary Heart):ti,ab,kw OR (Heart Disease*, Coronary):ti,ab,kw OR (Ischemia*, Myocardial):ti,ab,kw OR (Ischemic Heart Disease*):ti,ab,kw OR (Heart Disease*, Ischemic):ti,ab,kw OR (Disease*, Ischemic Heart):ti,ab,kw OR (Coronary Intervention*, Percutaneous):ti,ab,kw OR (Intervention*, Percutaneous Coronary):ti,ab,kw OR (Percutaneous Coronary Revascularization*):ti,ab,kw OR (Coronary Revascularization*, Percutaneous):ti,ab,kw OR (Revascularization*, Percutaneous Coronary):ti,ab,kw OR (Percutaneous Transluminal Coronary Angioplast):ti,ab,kw OR (Artery Bypass*, Coronary):ti,ab,kw OR (Coronary Artery Bypass Surgery):ti,ab,kw OR (Coronary Artery Bypass Grafting):ti,ab,kw OR (Bypass*, Aortocoronary):ti,ab,kw OR (Bypass Surgery, Coronary Artery):ti,ab,kw	43,218
#9	#1 OR #2 OR #3 OR #4 OR #5 OR #6 OR #7 OR #8	65,074
#10	MeSH descriptor: [Pharmaceutical Preparations] explode all trees	82,291
#11	MeSH descriptor: [Polypharmacy] explode all trees	405
#12	MeSH descriptor: [Prescription Drugs] explode all trees	149
#13	(medication* or regimen* or prescription* or prescribed* or drug* or pill* or tablet*):ti,ab,kw	841,009
#14	#10 or #11 or #12 or #13	852,920
#15	MeSH descriptor: [Patient Compliance] explode all trees	15,212
#16	MeSH descriptor: [Medication Adherence] explode all trees	3,255
#17	(adher* or non-adher* or nonadher* or complian* or non-complian* or noncomplian* or persisten* or compl* or concord*):ti,ab,kw	618,059
#18	#15 or #16 or #17	618,098
#19	MeSH descriptor: [Mortality] explode all trees	21,880
#20	(mortalit* or death* or fatalit*):ti,ab,kw	171,912
#21	((cardiovascular or cardiac) NEAR/3 (arrest* or dead* or death*)):ti,ab,kw	15,148
#22	#19 or #20 or #21	177,810
#23	MeSH descriptor: [Secondary Prevention] this term only	4,014
#24	(secondary NEAR/3 prevent*):ti,ab,kw	9,559
#25	(prevent* NEAR/3 recurrenc*):ti,ab,kw	4,771
#26	(surviv* or prognos*):ti,ab,kw	173,047
#27	#23 or #24 or #25 or #26	184,092
#28	animal*	44,706

#29	conference abstract or conference paper or conference review	244,658
#30	editorial or letter or case reports or comment or note	52,749
#31	#9 and #14 and #18 and #22 not #27 not #28 not #29 not #30	2,236

Literature search in Embase

No.	Search Details	Results
#1	'coronary artery disease'/exp OR 'acute coronary syndrome'/exp OR 'coronary artery bypass graft'/exp OR 'percutaneous coronary intervention'/exp OR 'coronary diseases':ab,ti OR 'disease*, coronary':ab,ti OR 'coronary heart diseases':ab,ti OR 'disease*, coronary heart':ab,ti OR 'heart disease*, coronary':ab,ti OR 'ischemia*, myocardial':ab,ti OR 'artery disease*, coronary':ab,ti OR 'left main coronary artery disease':ab,ti OR 'left main disease*':ab,ti OR 'coronary arterioscleros*':ab,ti	530,208
#2	'drug'/exp OR 'polypharmacy'/exp OR 'prescription drug'/exp	3,676,267
#3	'medication* OR regimen* OR prescription* OR prescribed* OR drug* OR pill* OR 'tablet'/exp OR tablet	14,376,466
#4	#2 OR #3	14,393,858
#5	'patient compliance'/exp OR 'medication compliance'/exp	190,189
#6	adher* OR 'non adher*' OR nonadher* OR complian* OR 'non complian*' OR noncomplian* OR persisten* OR compl* OR concord*	10,149,299
#7	#5 or #6	10,149,299
#8	'mortality'/exp	1,397,330
#9	mortalit* OR death* OR fatalit*	3,314,748
#10	#8 or #9	3,314,767
#11	'secondary prevention'/exp	34,689
#12	surviv* OR prognos*	3,368,165
#13	#11 or #12	3,397,305
#14	#1 and #4 and #7 and #10 and #13 AND [humans]/lim AND [embase]/lim AND [article]/lim AND [clinical study]/lim) NOT letter NOT comment NOT editorial NOT 'review' NOT 'meta analysis' NOT	3902

	'animal' NOT erratum NOT note	

Literature search in Web of Science

No.	Search Details	Results
#1	TS=(Coronary Disease* OR myocardial ischemia OR Coronary Artery Disease OR Acute Coronary Syndrome OR Percutaneous Coronary Revascularization OR percutaneous coronary intervention OR coronary artery bypass OR Myocardial Revascularization OR Disease*, Coronary OR Coronary Heart Disease* OR Ischemia*, Myocardial OR Ischemic Heart Disease* OR Coronary Artery Disease* OR Left Main Coronary Artery Disease OR Left Main Disease* OR Left Main Coronary Disease OR Coronary Arterioscleros* OR Coronary Atheroscleros* OR Coronary Syndrome*, Acute OR Coronary Intervention*, Percutaneous OR Percutaneous Coronary Revascularization* OR Percutaneous Transluminal Coronary Angioplast* OR Artery Bypass*, Coronary OR Coronary Artery Bypass Surgery OR Coronary Artery Bypass Grafting OR Aortocoronary Bypass* OR Bypass Surgery, Coronary Artery OR Internal Mammary Artery Implantation OR Revascularization*, Myocardial)	1,040,527
#2	TS=（Pharmaceutical Preparations OR polypharmacy OR Prescription Drugs OR medication* OR regimen* OR prescription* OR prescribed* OR drug* OR pill* OR tablet*）	11,982,647
#3	TS=（Patient Compliance OR Medication Adherence OR adher* OR non-adher* OR nonadher* OR complian* OR non-complian* OR noncomplian* OR persisten* OR compl* OR concord*）	18,016,787
#4	TS=（Mortality OR mortalit* OR death* OR fatalit*）	3,528,656
#5	TS=（Secondary Prevention OR surviv* OR prognos*）	3,829,377
#6	TS=（animal*）	22,273,770
#7	#1 AND #2 AND #3 AND #4 AND #5 NOT #6	4,762
#8	#7 and Abstract or Meeting or Unspecified or 综述论文 or 社论材料 or Case Report or Patent or Reference Material or 信函 or News or 收回的出版物 or 书籍 or Biography or 修订 (排除 – 文献类型) and Cardiovascular System Cardiology (研究方向)	2,460

b. Figure S1. PRISMA 2020 Flow Diagram for new systematic reviews

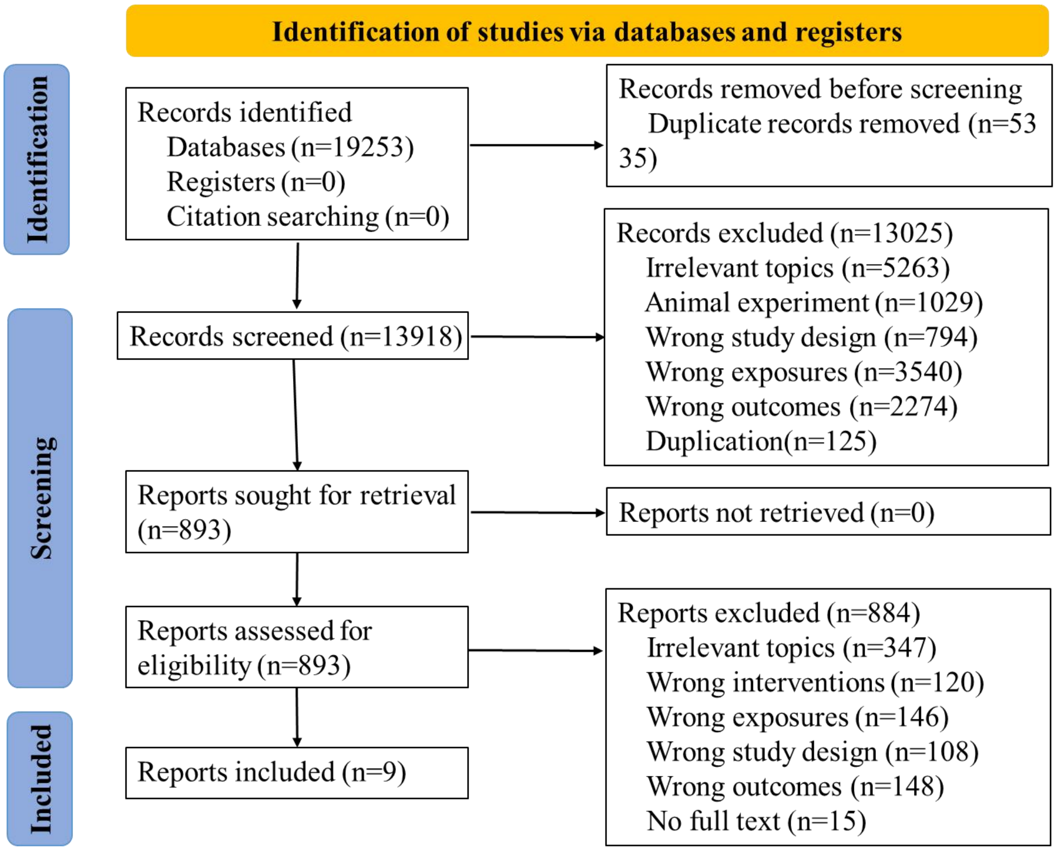

c. Table S1. Measurement and definition of exposures of included studies reporting on the association of medication adherence trajectories with clinical outcomes in patients with CVD.

**First Author**	**Definition of medication adherence trajectories**	**Maximum number of medication adherence assessments, years of follow-up**	**% of patients lost from first to second measurement due to events.**	**Reverse causation analysis**

Hickson et al., 2019	A “major decrease” if patients were adherent pre-AMI and severely nonadherent post-AMI, a “moderate decrease” for all other adherence decreases, “no change,” a “major increase” if patients were severely nonadherent pre-AMI and adherent post-AMI, and a “moderate increase” for all other adherence increases.	2, 1.5	14.1/0.93	No
Kumbhani et al., 2013	consistent adherers (fully adherent at baseline and at 1 year); negative converters (fully adherent at baseline, but not at 1 year); positive converters (nonadherent at baseline, but fully adherent at 1 year); and consistent nonadherers (nonadherent at both baseline and 1 year).	2, 4	7.2	No
May et al., 2022	fully adherent, defined as PDC ≥80% for Years 1–5 or until death; short-term-adherent, defined as PDC ≥80% for Years 1–3 only; early-adherent only, defined as PDC ≥80% for Year 1 only; complex-adherent, defined as PDC ≥80% in any of Years 2–5, but not Year 1; or non-adherent, defined as PDC <80% for Years 1–5 or until death.	5, 5	36.4	No

Rodríguez-Bernal et al., 2022	1. Adherent: Patients consistently adhered to their medications throughout the first year.<br>2. Early Gap: Patients started with good adherence but experienced gaps in their medication regimen early in the year.<br>3. Middle Gap: Patients had good adherence initially but had interruptions in their regimen around the middle of the year.<br>4.Late Decline: Patients maintained good adherence for the initial months but showed a decline in adherence later in the year.<br>5. Occasional Users: Patients exhibited sporadic adherence with no consistent pattern throughout the year.<br>6. Early Decline: Patients showed a decline in adherence shortly after the start and continued with poor adherence.<br>7. Non-Adherent: Patients had consistently low adherence or were non-adherent throughout the year.	12, 2	5.9	Yes. The study excluded patients who died within the first year after discharge from the analysis. Since the first year was used to measure adherence, including these patients could bias the results due to early death unrelated to adherence patterns.

Turgeon et al., 2022	1. early consistent non-adherence: patients either never initiated or promptly stopped P2Y12 inhibitor use within the first month.<br>2.rapid decline: discontinued P2Y12 inhibitor use after persisting for 3 months<br>3. delayed initiation: poor initial P2Y12 inhibitor adherence that improved over the study period.<br>4. gradual decline: high initial P2Y12 inhibitor adherence that steadily declined.<br>5. persistent adherence: high P2Y12 inhibitor adherence throughout the study period.	12, 1	1	Yes. 1. This study excludes patients who were lost to follow-up or died during the initial hospitalization, ensuring that the analysis focuses on those with complete data for the follow-up period.<br>2. Temporal Separation of Adherence and Outcomes: The study design involves measuring medication adherence trajectories in the first 12 months post-discharge and then analyzing the association of these trajectories with clinical outcomes (MACE and major bleeding) over the same period. By identifying adherence patterns first and then examining their impact on outcomes, the study helps ensure that the observed clinical outcomes are a consequence of the adherence patterns rather than the other way around.

An et al., 2022	1. Consistent Adherence: Patients in this group maintained high levels of adherence throughout the 3.5 years.<br>2. Early Discontinuation: Patients in this group discontinued DOAC therapy within the first 6 months.<br>3. Gradually Declining Adherence: Patients initially adhered well to DOACs, but their adherence declined gradually over time.	monthly, 3.5	1.2	Yes, patients who experienced significant early events (within the first 30 days) were excluded from the adherence analysis, as these events could influence subsequent adherence patterns.
Kang et al., 2023	1. Consistently high adherence: stable, high levels of medication adherence over time.<br>2. Gradually declining adherence: shows a slow decrease in medication adherence.<br>3. Rapidly declining adherence: a quick drop in adherence.<br>4.No extended treatment group: includes patients who did not continue warfarin treatment beyond the initial period	Monthly, 6 months	29.7	Yes, patients who did not complete the initial 6-month anticoagulant treatment without developing recurrent VTE or major bleeding were excluded from the final analysis

Kang et al., 2023	1. Consistently high adherence: stable, high levels of medication adherence over time.<br>2. Gradually declining adherence: shows a slow decrease in medication adherence.<br>3. Rapidly declining adherence: a quick drop in adherence.<br>4.No extended treatment group: includes patients who did not continue warfarin treatment beyond the initial period	Monthly, 6 months	29.7	Yes, patients who did not complete the initial 6-month anticoagulant treatment without developing recurrent VTE or major bleeding were excluded from the final analysis
Park et al., 2023	1. Consistently High Adherence: Patients who maintained a high level of medication adherence throughout the extended treatment period.<br>2. Gradually Declining Adherence: Patients whose adherence decreased gradually over time.<br>3. Rapidly Declining Adherence: Patients whose adherence decreased rapidly after the initial treatment period.<br>4. No Extended Treatment: Patients who did not continue with the extended treatment after the initial 6-month therapy period	15-day intervals, 6 months	1.62	No, Reverse causality between adherence and outcomes (eg, major bleeding) may exist.

Supplementary section 2: Assessment of quality and risk of bias results

a. Figure S2. Funnel plots for all-cause mortality

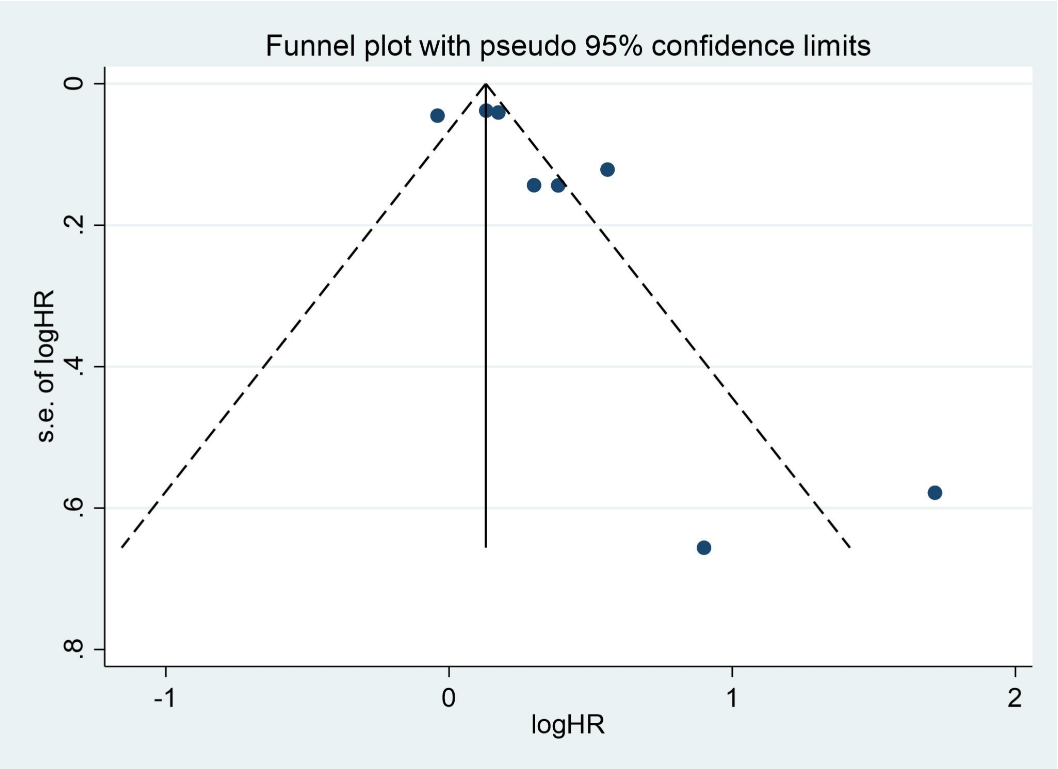

b. Figure S3. Funnel plots for MACE incidence rate

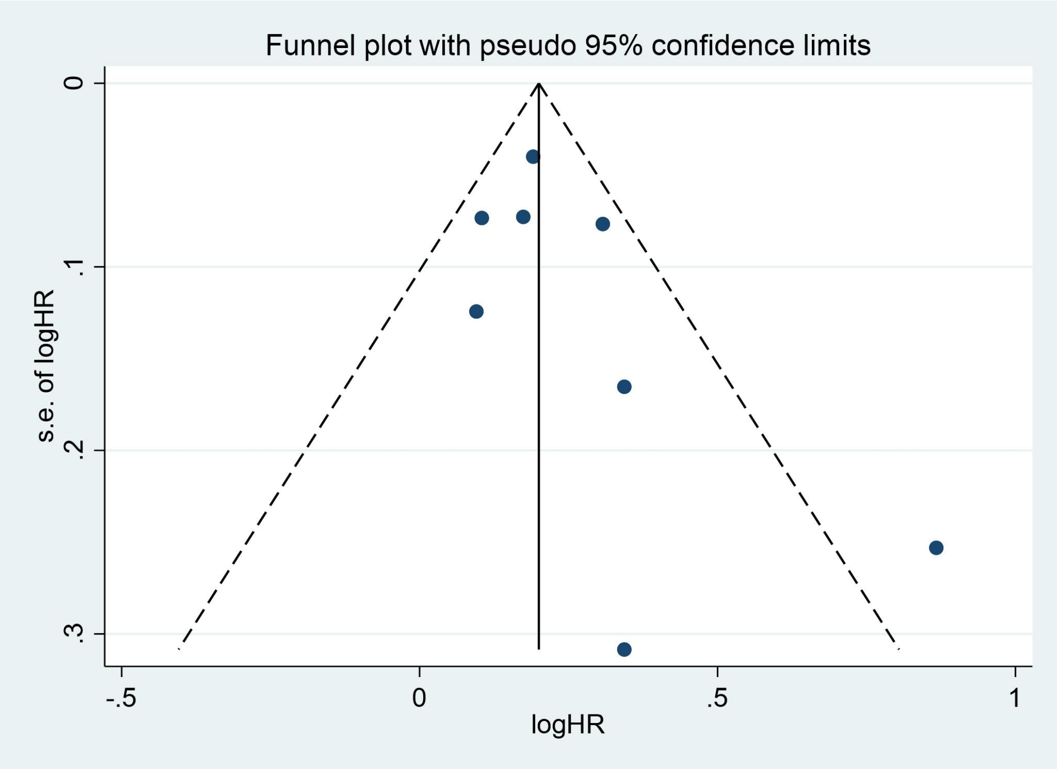

c. Figure S4. Funnel plots for Recurrent Venous Thromboembolism

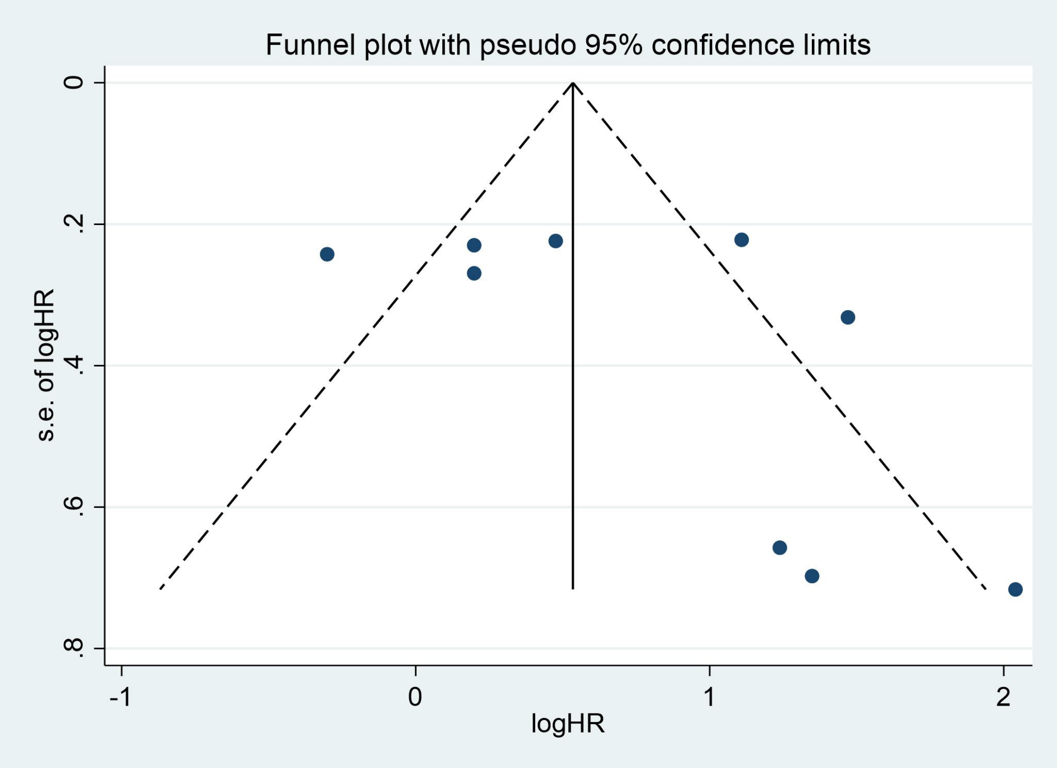

d. Figure S5. Funnel plots for major bleeding

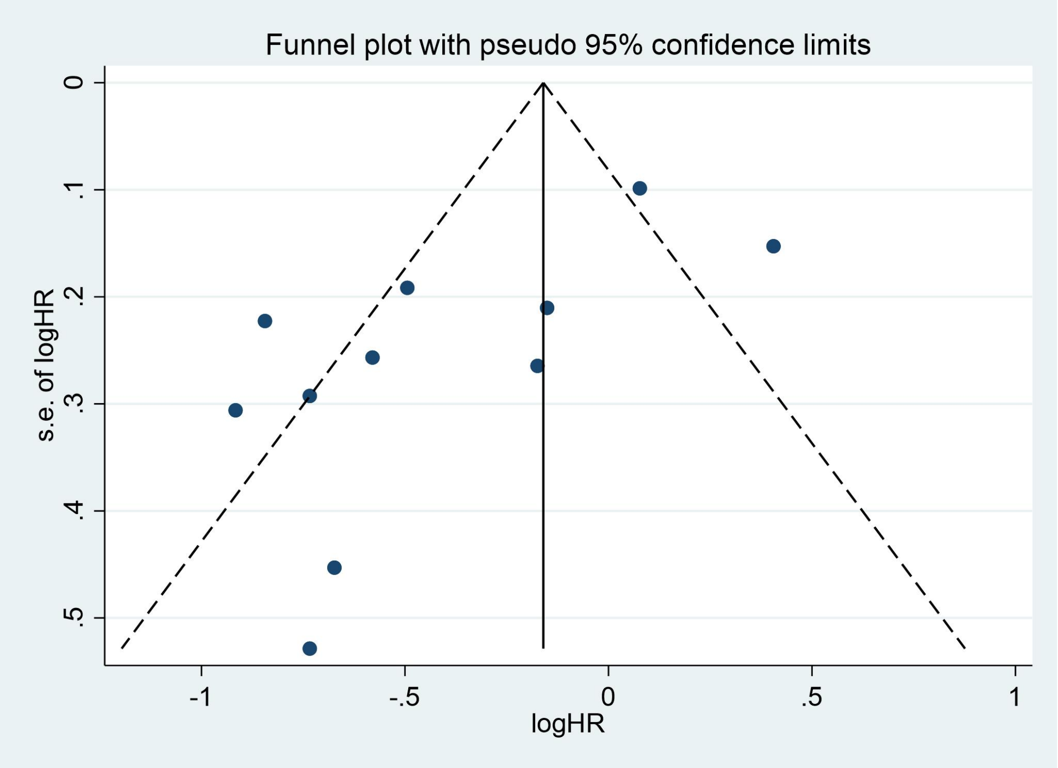

e. Egger test for small-study effects in studies reporting all-cause mortality

Begg's Test

```
  adj. Kendall's Score (P-Q) =       6
          Std. Dev. of Score =    8.08
           Number of Studies =       8
                          z  =    0.74
                  Pr > |z| =   0.458
                          z  =    0.62 (continuity corrected)
                  Pr > |z| =   0.536 (continuity corrected)
```

Egger's test

Std_Eff	Coefficient	Std. err.	t	P>|t|	[95% conf.	interval]
slope	.0019225	.0696866	0.03	0.979	-.1685943	.1724394
bias	2.545297	1.09151	2.33	0.058	-.1255314	5.216126

f. Egger test for small-study effects in studies reporting MACE incidence rate

Begg's Test

```
  adj. Kendall's Score (P-Q) =       8
          Std. Dev. of Score =    8.08
           Number of Studies =       8
                          z  =    0.99
                  Pr > |z| =   0.322
                          z  =    0.87 (continuity corrected)
                  Pr > |z| =   0.386 (continuity corrected)
```

Egger's test

Std_Eff	Coefficient	Std. err.	t	P>|t|	[95% conf.	interval]
slope	.1206599	.0660043	1.83	0.117	-.0408469	.2821667
bias	1.189403	.8407532	1.41	0.207	-.8678461	3.246652

g. Egger test for small-study effects in studies reporting Recurrent Venous Thromboembolism

Begg's Test

```
  adj. Kendall's Score (P-Q) =       8
          Std. Dev. of Score =    9.59
           Number of Studies =       9
                          z  =    0.83
                  Pr > |z| =   0.404
                          z  =    0.73 (continuity corrected)
                  Pr > |z| =   0.466 (continuity corrected)
```

Egger's test

Std_Eff	Coefficient	Std. err.	t	P>|t|	[95% conf.	interval]
slope	-.1689813	.5219296	-0.32	0.756	-1.403149	1.065186
bias	2.600032	1.786084	1.46	0.189	-1.623385	6.823448

h. Egger test for small-study effects in studies reporting Major bleeding

```
Begg's Test

  adj. Kendall's Score (P-Q) =      -15
          Std. Dev. of Score =    12.85
           Number of Studies =       11
                          z  =    -1.17
                    Pr > |z| =    0.243
                          z  =     1.09 (continuity corrected)
                    Pr > |z| =    0.276 (continuity corrected)

Egger's test
```

Std_Eff	Coefficient	Std. err.	t	P>|t|	[95% conf.	interval]
slope	.445333	.2234578	1.99	0.077	-.0601636	.9508295
bias	-3.367908	1.119376	-3.01	0.015	-5.900111	-.8357045

i. Table S2. Assessment of quality and risk of bias according to the Newcastle-Ottawa scale.

**Author, ref**	**Selection /4**	**Comparability /2**	**Outcome /3**	**Total /9**	**Quality assessment**	**Risk of bias**
An 2021	3	2	3	8	Good	Low
Hickson 2019	4	2	3	9	Good	Low
Kang 2023	4	1	3	8	Good	Low
Kang 2023 DOAC	4	1	3	8	Good	Low
Kumbhani 2013	3	2	2	7	Fair	Moderate
May 2022	3	1	3	7	Fair	Moderate
Park 2023	4	2	3	9	Good	Low
Rodriguze 2022	4	1	3	8	Good	Low
Turgeon 2022	4	2	3	9	Good	Low

Thresholds for converting the Newcastle-Ottawa scales to AHRQ standards (good, fair, and poor): Good quality: 3 or 4 stars in selection domain AND 1 or 2 stars in comparability domain AND 2 or 3 stars in outcome/exposure domain. Fair quality: 2 stars in selection domain AND 1 or 2 stars in comparability domain AND 2 or 3 stars

in outcome/exposure domain. Poor quality: 0 or 1 star in selection domain OR 0 stars in comparability domain OR 0 or 1 stars in outcome/exposure domain.

Supplementary section 3: Medication adherence trajectories and major bleeding incidence rate

Figure S6. Meta-analysis on the associations of medication adherence trajectories with the risk of major bleeding incidence rate.

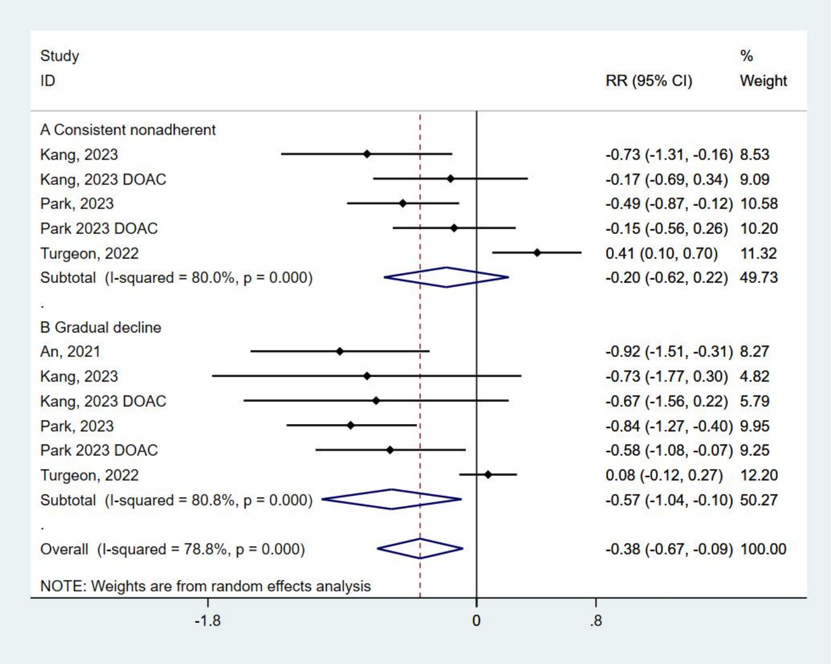

Supplementary section 4: Sensitivity analyses

Figure S7. Sensitivity analysis given named study is omitted.

a. consistent nonadherent. All-cause mortality

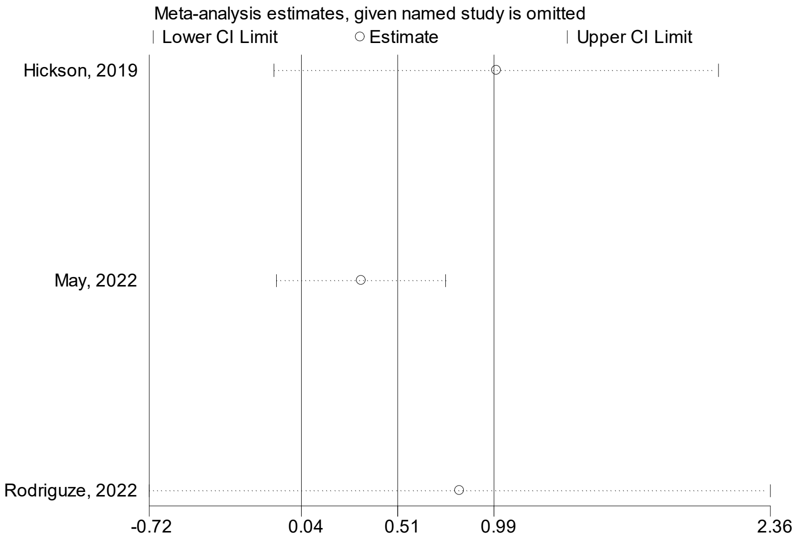

b. gradual decline. All-cause mortality

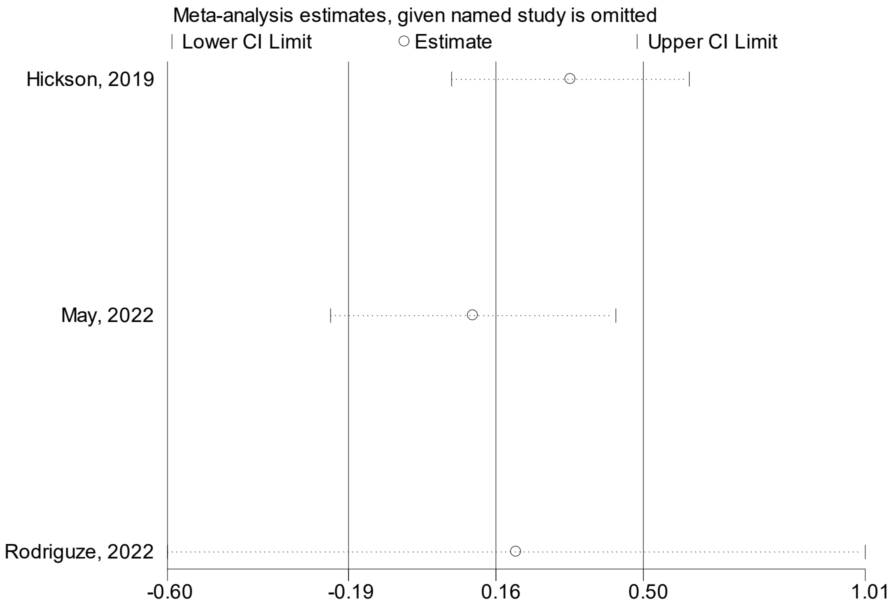

c. gradual increase. All-cause mortality

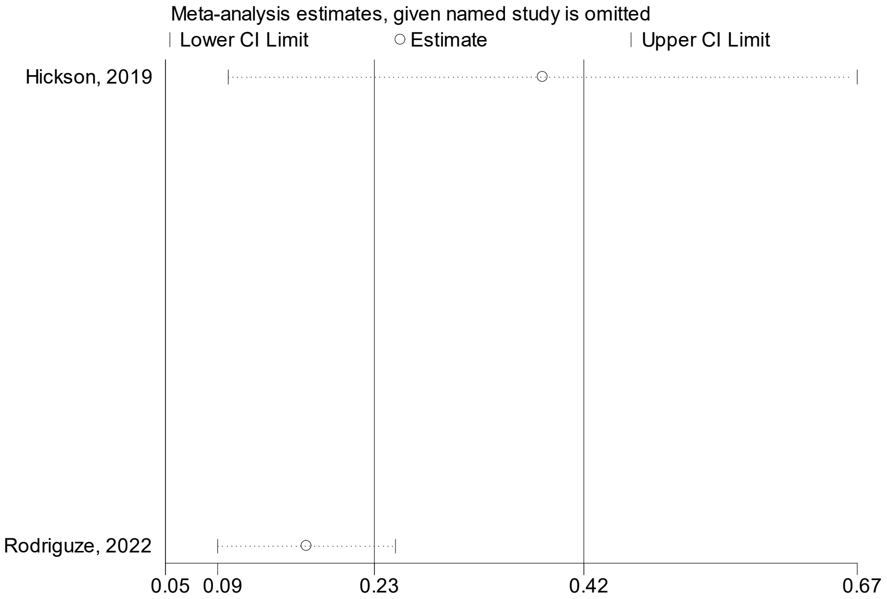

d. consistent nonadherent. MACE incidence rate

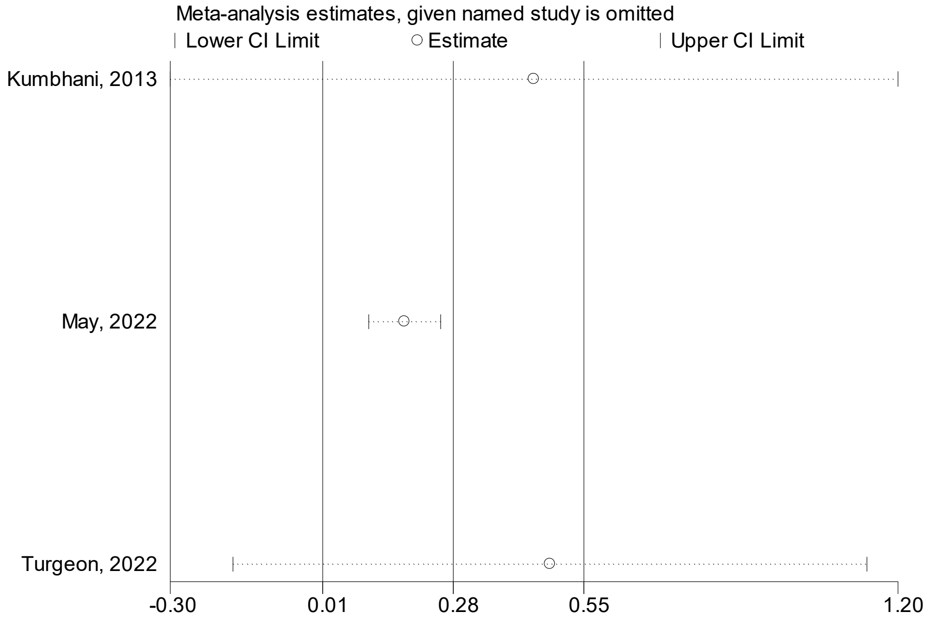

e. gradual decline. MACE incidence rate

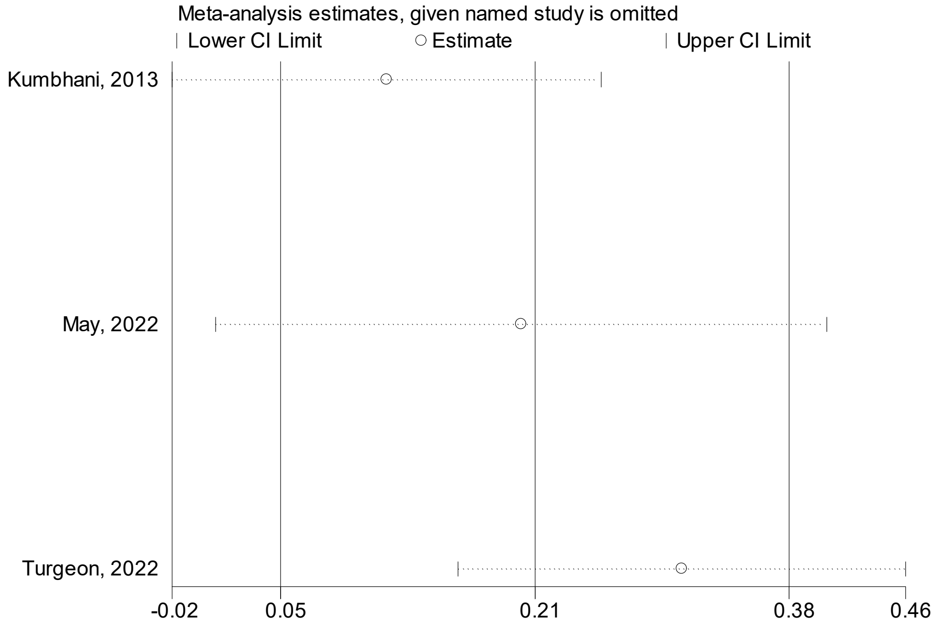

f. gradual increase. MACE incidence rate

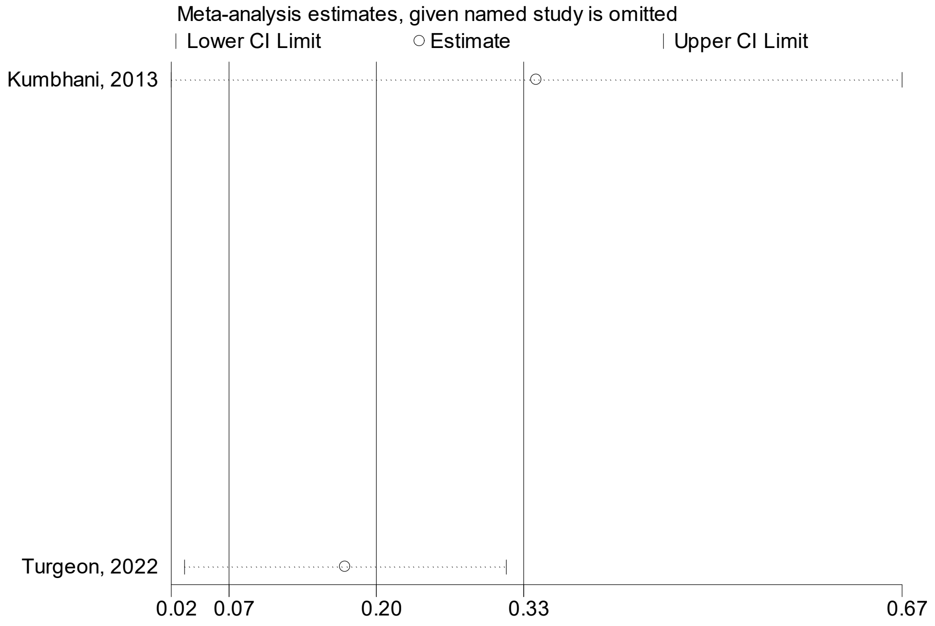

g. consistent nonadherent. Recurrent Venous Thromboembolism

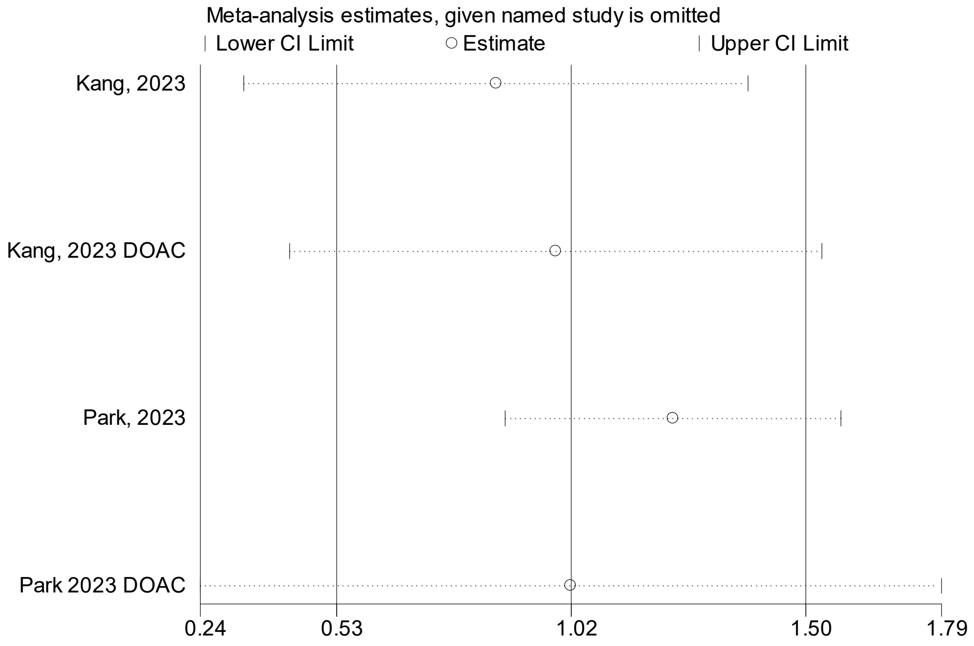

h. gradual decline. Recurrent Venous Thromboembolism

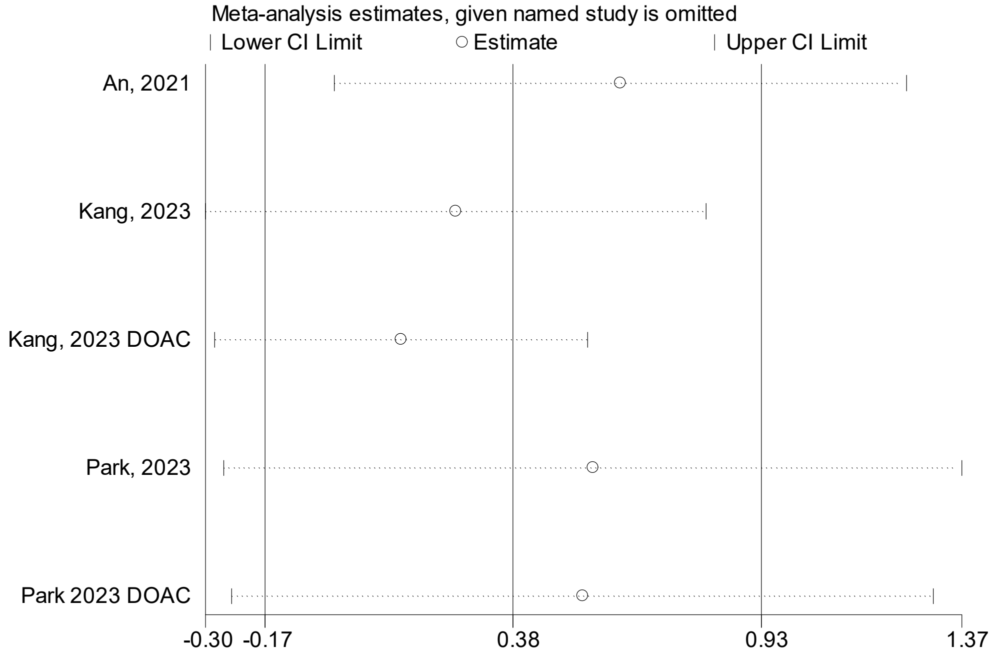

i. consistent nonadherent. Major bleeding

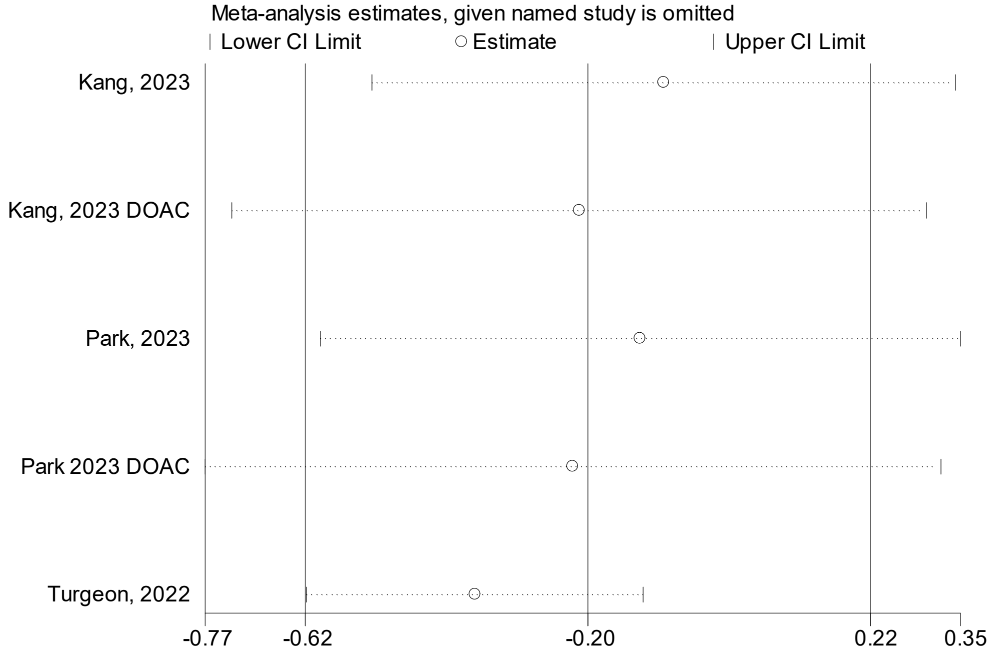

j. gradual decline. Major bleeding

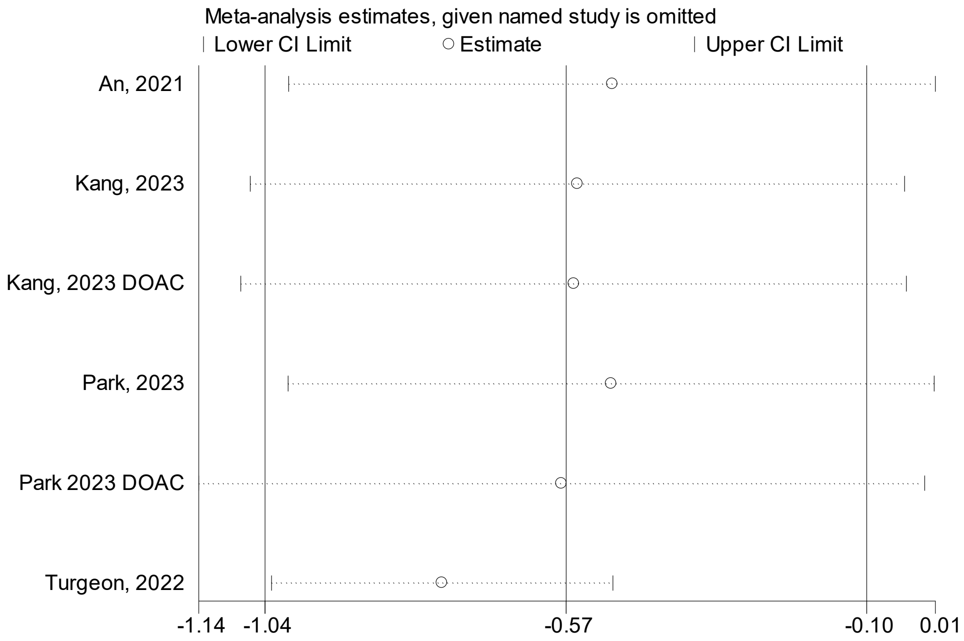

Figure S8. Random-effects meta-analysis of Recurrent Venous Thromboembolism according to age.

a. Random-effects meta-analysis of Recurrent Venous Thromboembolism in cohorts with mean age <65 years.

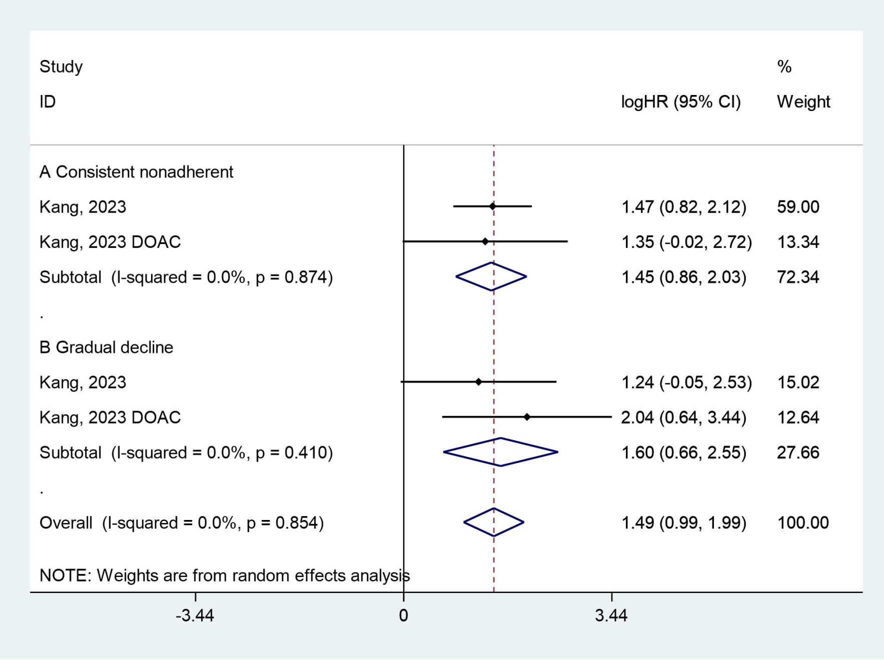

b. Random-effects meta-analysis of Recurrent Venous Thromboembolism in cohorts with mean age ≥65 years.

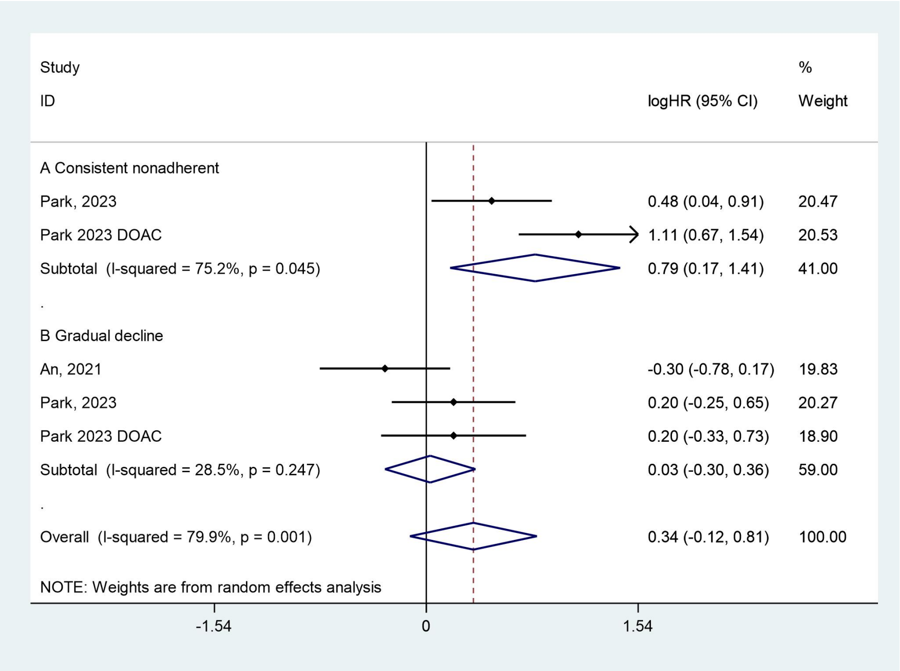

Figure S9. Random-effects meta-analysis of major bleeding according to age.

a. Random-effects meta-analysis of major bleeding in cohorts with mean age <65 years.

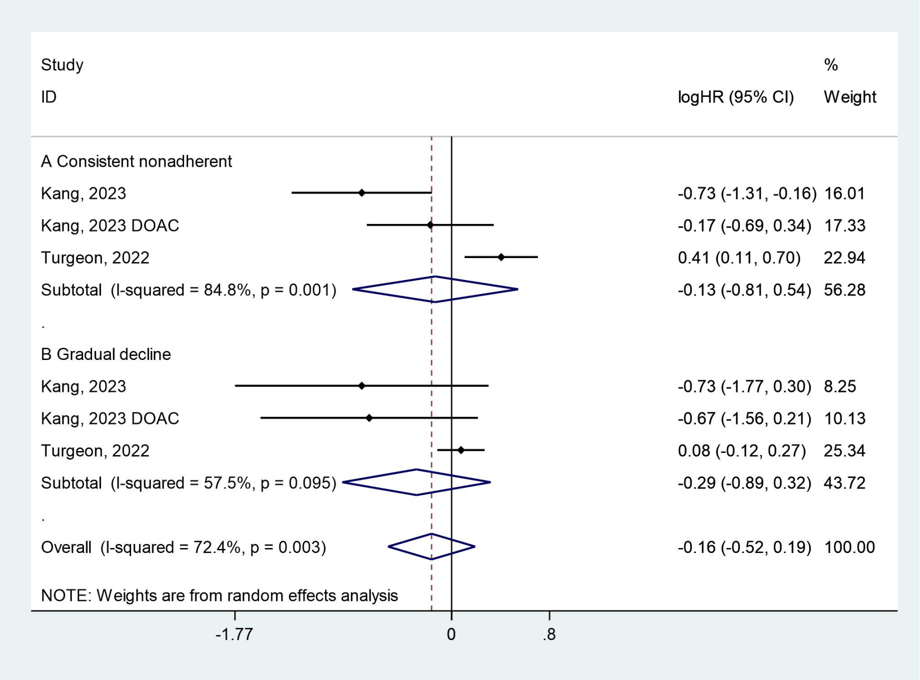

b. Random-effects meta-analysis of major bleeding in cohorts with mean age ≥65 years.

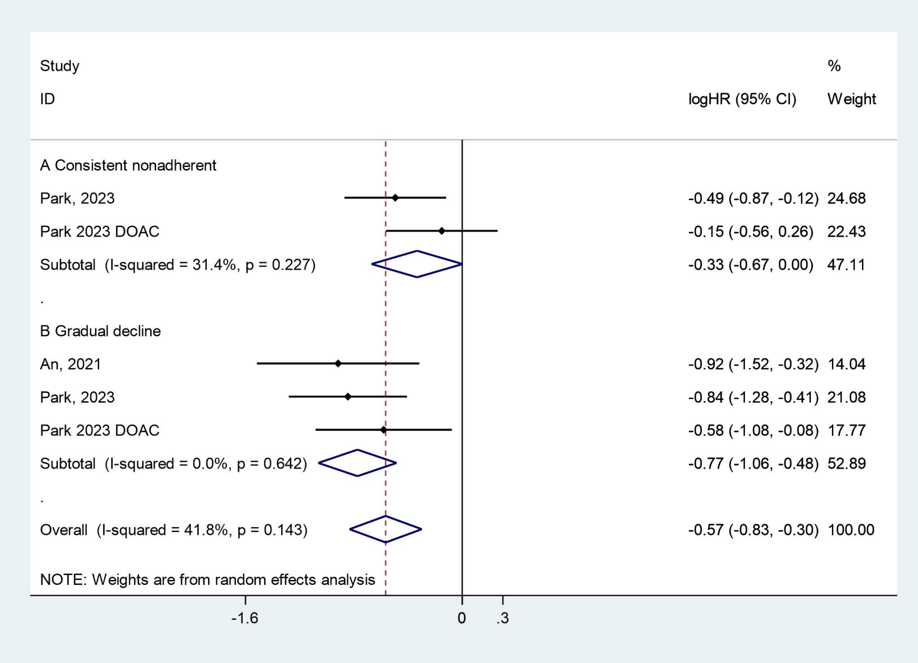

Figure S10. Meta-regressions to explore potential sources of heterogeneity.

a. all-cause mortality

```
  Effect-size label: Effect size
        Effect size: logHR
          Std. err.: se_logHR

Random-effects meta-regression                     Number of obs  =          8
Method: REML                                       Residual heterogeneity:
                                                               tau2 = .009452
                                                             I2 (%) =   70.99
                                                                 H2 =    3.45
                                                     R-squared (%) =   80.13
                                                   Wald chi2(2)    =   15.72
                                                   Prob > chi2     =  0.0004
```

_meta_es	Coefficient	Std. err.	z	P>|z|	[95% conf. interval]	
female	-.0070758	.0057648	-1.23	0.220	-.0183747	.004223
timeoffollowupyears	.3000419	.1556563	1.93	0.054	-.0050388	.6051226
diseasetype	0	(omitted)				
medication	0	(omitted)				
agecohorts	0	(omitted)				
_cons	.0239094	.4891003	0.05	0.961	-.9347096	.9825284

Test of residual homogeneity: Q_res = chi2(5) = 16.67   Prob > Q_res = 0.0052

b. MACE

Effect-size label: Effect size
Effect size: **logHR**
Std. err.: **se_logHR**

Random-effects meta-regression — Number of obs = **8**
Method: REML — Residual heterogeneity:
tau2 = **1.4e-07**
I2 (%) = **0.00**
H2 = **1.00**
R-squared (%) = **50.85**
Wald chi2(**2**) = **6.80**
Prob > chi2 = **0.0334**

_meta_es	Coefficient	Std. err.	z	P>|z|	[95% conf.	interval]
female	**-.0375167**	**.0178024**	**-2.11**	**0.035**	**-.0724087**	**-.0026247**
Timeoffollowupyears	**.1299813**	**.050687**	**2.56**	**0.010**	**.0306366**	**.229326**
diseasetype	**0**	(omitted)				
medication	**0**	(omitted)				
agecohorts	**0**	(omitted)				
_cons	**.8882366**	**.3883105**	**2.29**	**0.022**	**.127162**	**1.649311**

Test of residual homogeneity: Q_res = chi2(**5**) = **5.68** Prob > Q_res = **0.3384**

c. recurrent VTE

Effect-size label: Effect size
Effect size: **logHR**
Std. err.: **se_logHR**

Random-effects meta-regression — Number of obs = **9**
Method: REML — Residual heterogeneity:
tau2 = **.1046**
I2 (%) = **55.68**
H2 = **2.26**
R-squared (%) = **72.04**
Wald chi2(**3**) = **13.28**
Prob > chi2 = **0.0041**

_meta_es	Coefficient	Std. err.	z	P>|z|	[95% conf.	interval]
female	**-.1661065**	**.3969678**	**-0.42**	**0.676**	**-.9441491**	**.611936**
timeoffollowupyears	**-1.155444**	**2.122494**	**-0.54**	**0.586**	**-5.315455**	**3.004568**
diseasetype	**0**	(omitted)				
medication	**0**	(omitted)				
agecohorts	**.4656243**	**3.502618**	**0.13**	**0.894**	**-6.39938**	**7.330629**
_cons	**9.921058**	**17.44823**	**0.57**	**0.570**	**-24.27685**	**44.11896**

Test of residual homogeneity: Q_res = chi2(**5**) = **11.01** Prob > Q_res = **0.0511**

d. Major bleeding

```
Effect-size label: Effect size
      Effect size: logHR
        Std. err.: se_logHR

Random-effects meta-regression                    Number of obs  =         11
Method: REML                                      Residual heterogeneity:
                                                              tau2 =  .03033
                                                            I2 (%) =   40.79
                                                                H2 =    1.69
                                                     R-squared (%) =   80.06
                                                  Wald chi2(4)     =   19.16
                                                  Prob > chi2      =  0.0007
```

_meta_es	Coefficient	Std. err.	z	P>|z|	[95% conf.	interval]
female	-.2415381	.2313524	-1.04	0.296	-.6949805	.2119042
timeoffollowupyears	-.5413146	.2883652	-1.88	0.060	-1.1065	.0238709
diseasetype	2.649767	2.956979	0.90	0.370	-3.145806	8.445341
medication	0	(omitted)				
agecohorts	2.207392	2.125217	1.04	0.299	-1.957957	6.372741
_cons	1.601823	.7193067	2.23	0.026	.1920078	3.011638

```
Test of residual homogeneity: Q_res = chi2(6) =   9.41    Prob > Q_res = 0.1517
```

Figure S11. Cumulative random-effects meta-analysis of all-cause mortality according to year of publication.

a. Consistent nonadherent

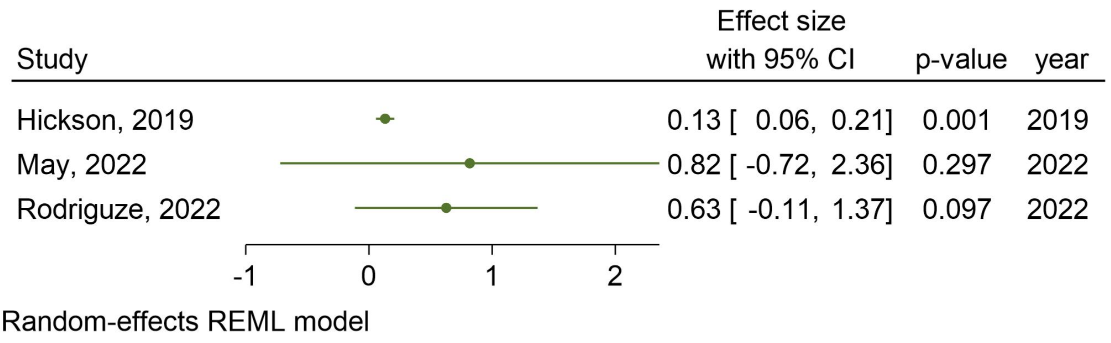

b. Gradual decline

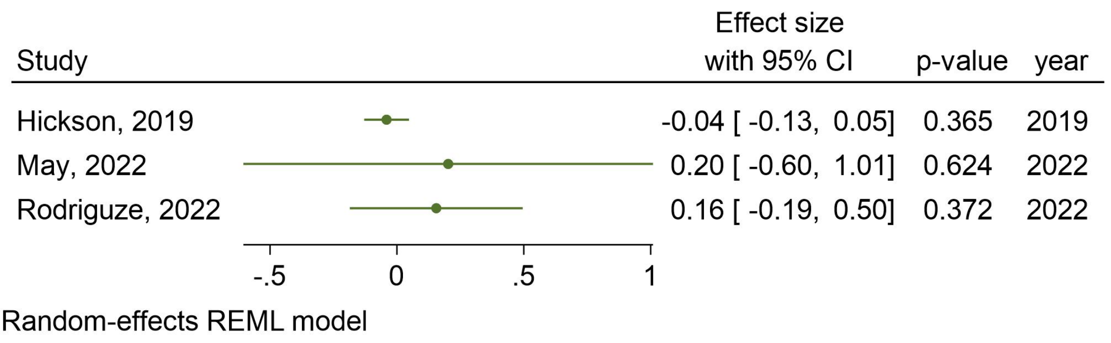

c. Gradual increase

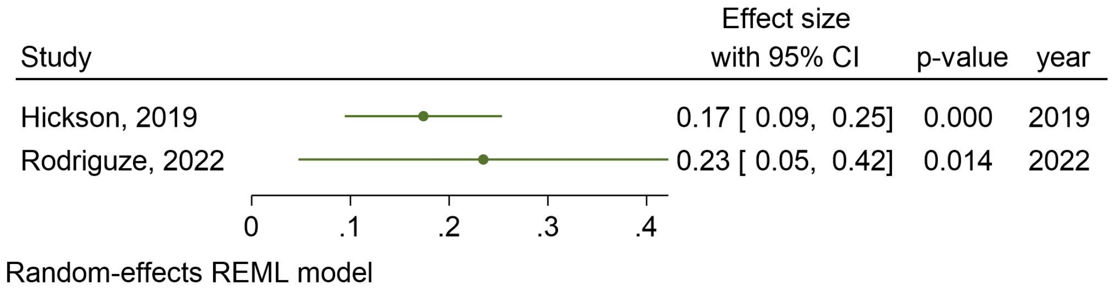

Figure S12. Cumulative random-effects meta-analysis of MACE incidence rate according to year of publication.

a. Consistent nonadherent

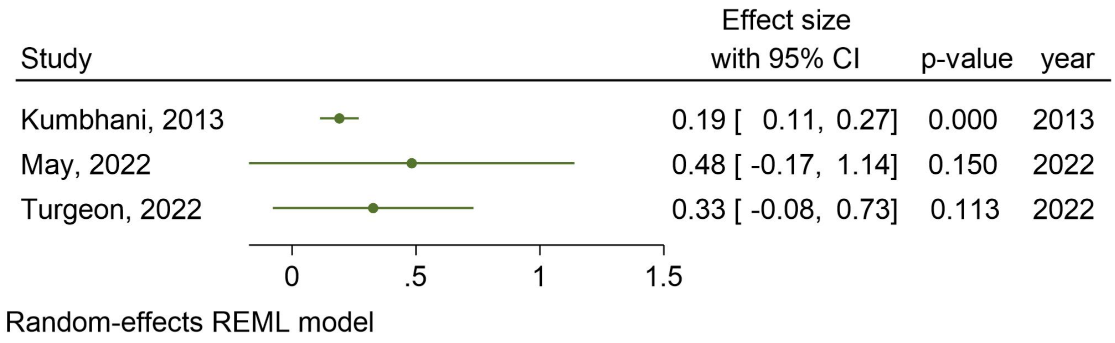

b. Gradual decline

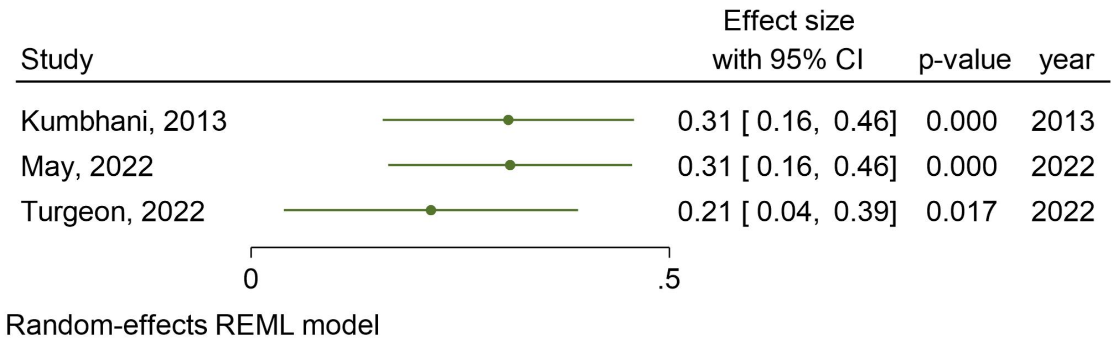

c. Gradual increase

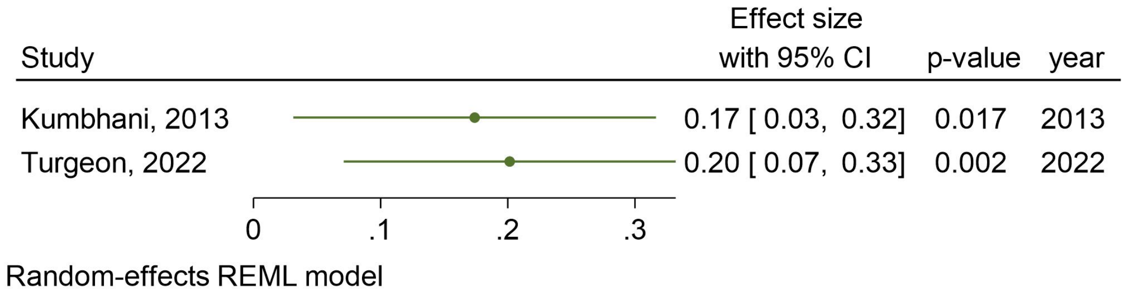

Figure S13. Cumulative random-effects meta-analysis of Recurrent Venous Thromboembolism incidence rate according to year of publication.

a. Consistent nonadherent

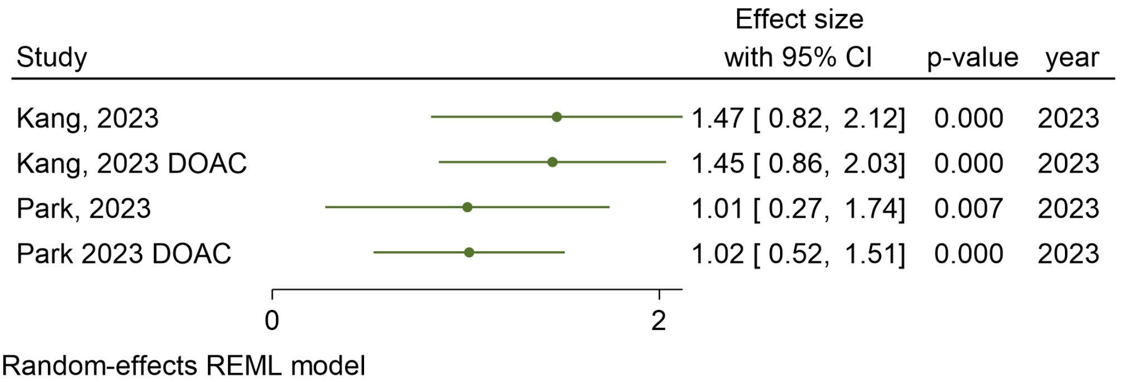

b. Gradual decline

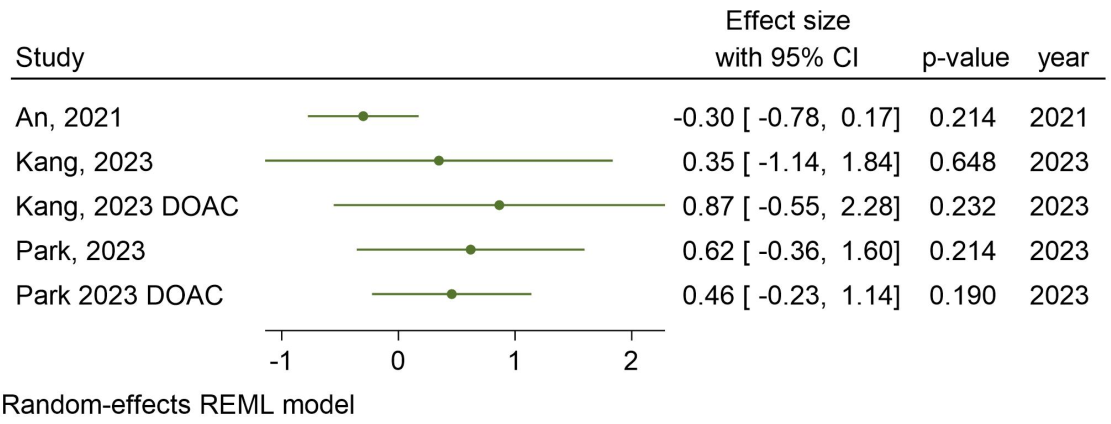

Figure S14. Cumulative random-effects meta-analysis of major bleeding incidence rate according to year of publication.

a. Consistent nonadherent

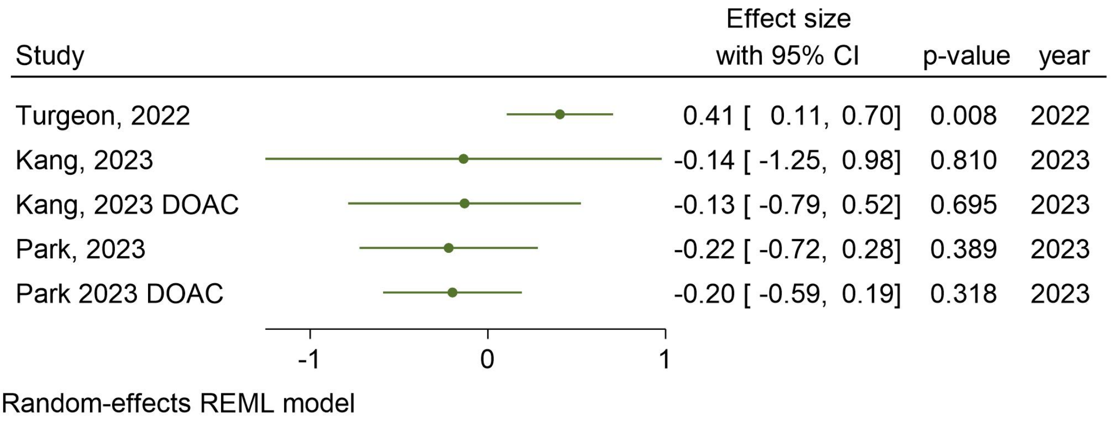

b. Gradual decline

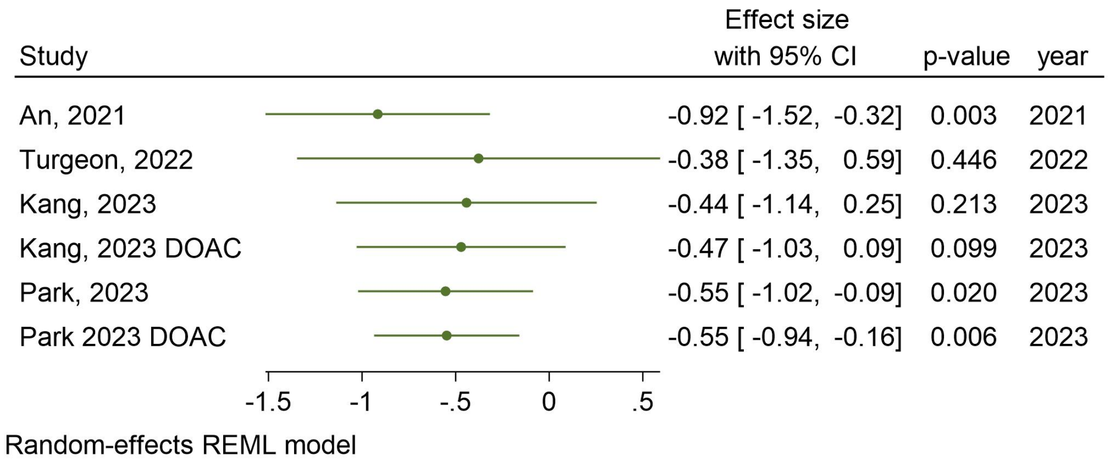

Figure S15. Cumulative random-effects meta-analysis of all-cause mortality according to time of follow up.

a. Consistent nonadherent

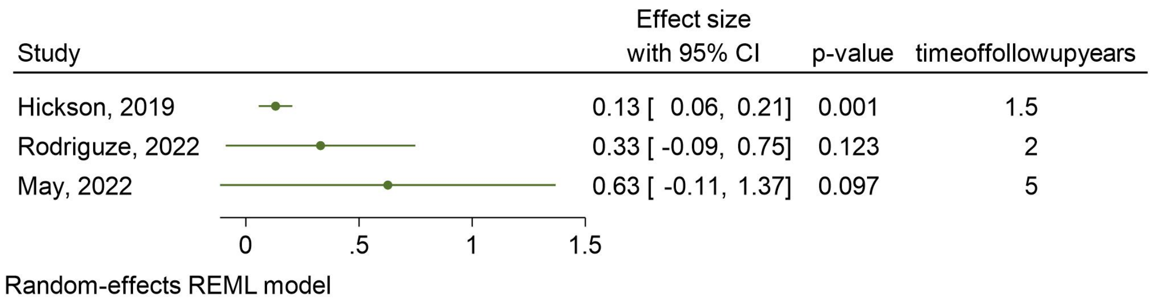

b. Gradual decline

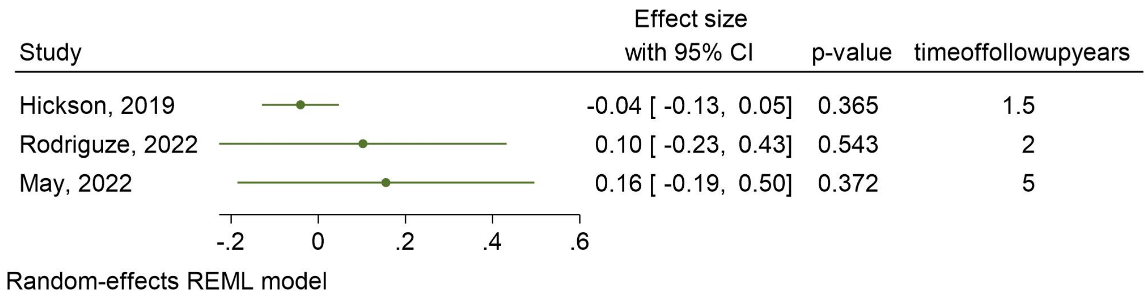

c. Gradual increase

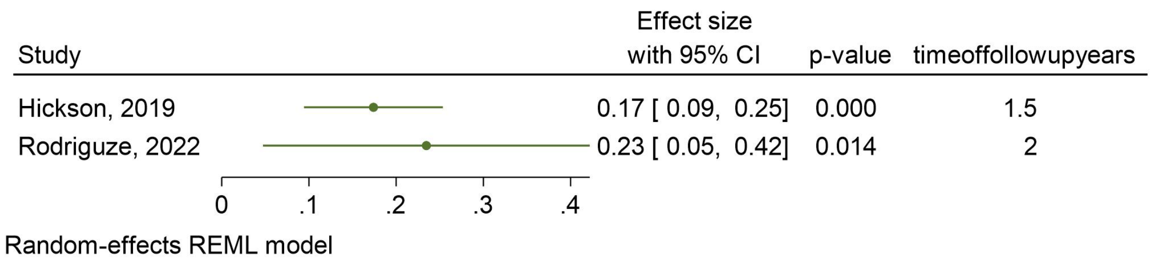

Figure S16. Cumulative random-effects meta-analysis of MACE incidence rate according to time of follow up.

a. Consistent nonadherent

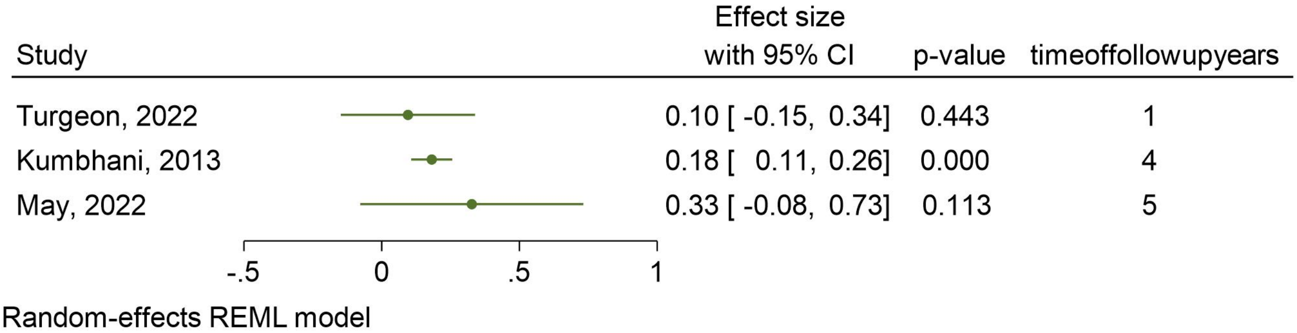

b. Gradual decline

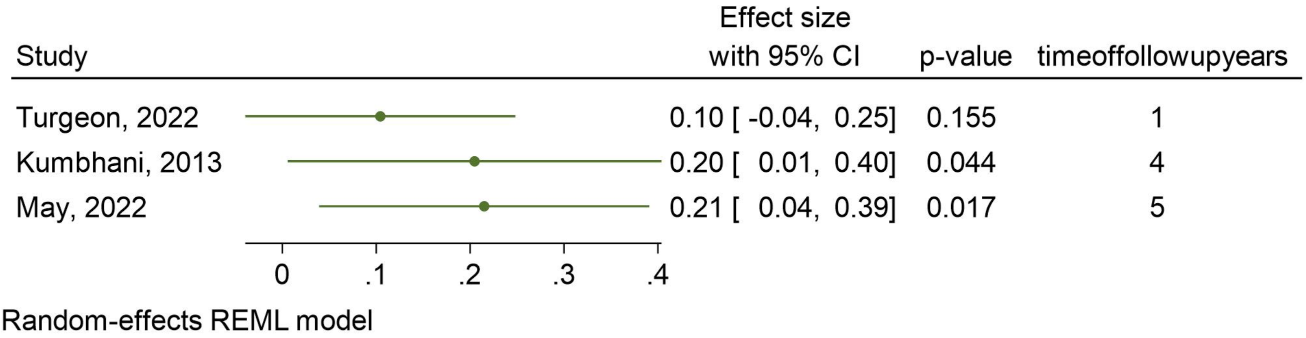

c. Gradual increase

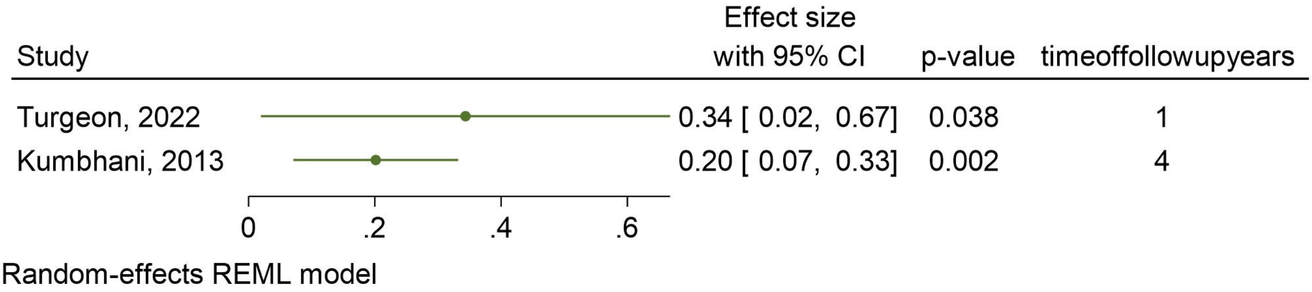

Figure S17. Cumulative random-effects meta-analysis of Recurrent Venous Thromboembolism according to time of follow up.

a. Consistent nonadherent

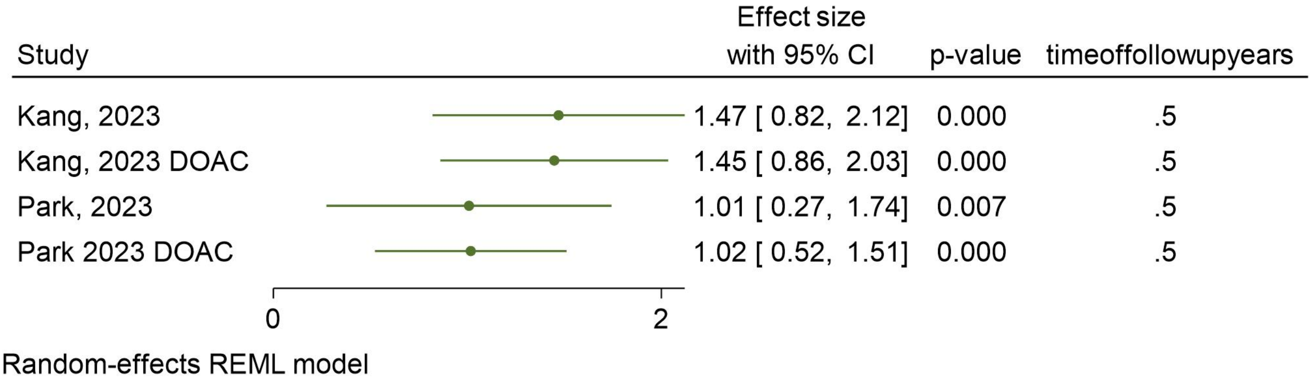

b. Gradual decline

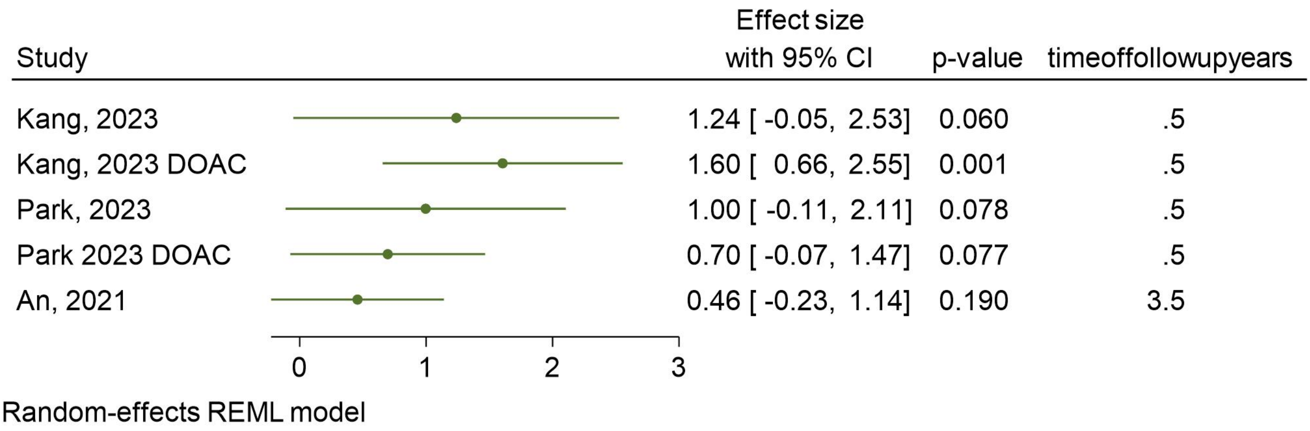

Figure S18. Cumulative random-effects meta-analysis of major bleeding according to time of follow up.

a. Consistent nonadherent

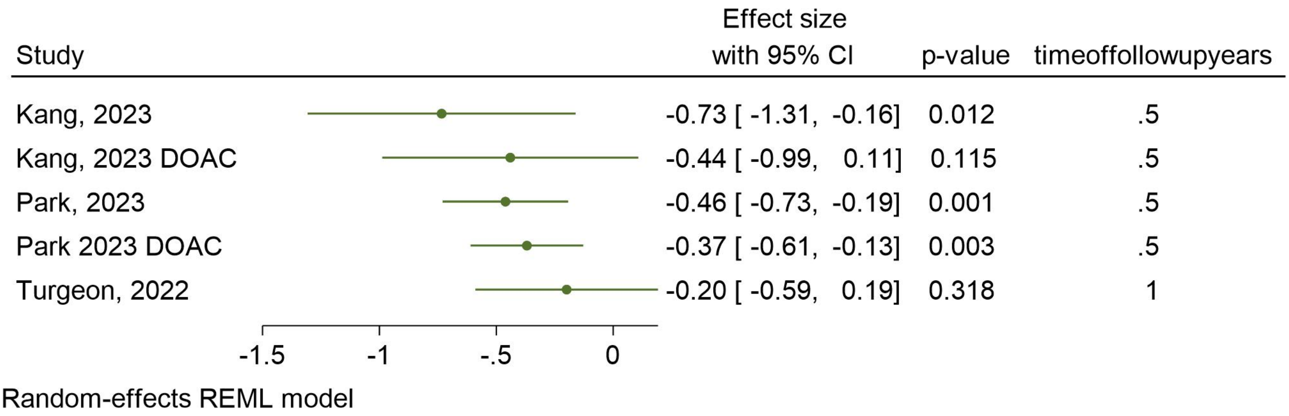

b. Gradual decline

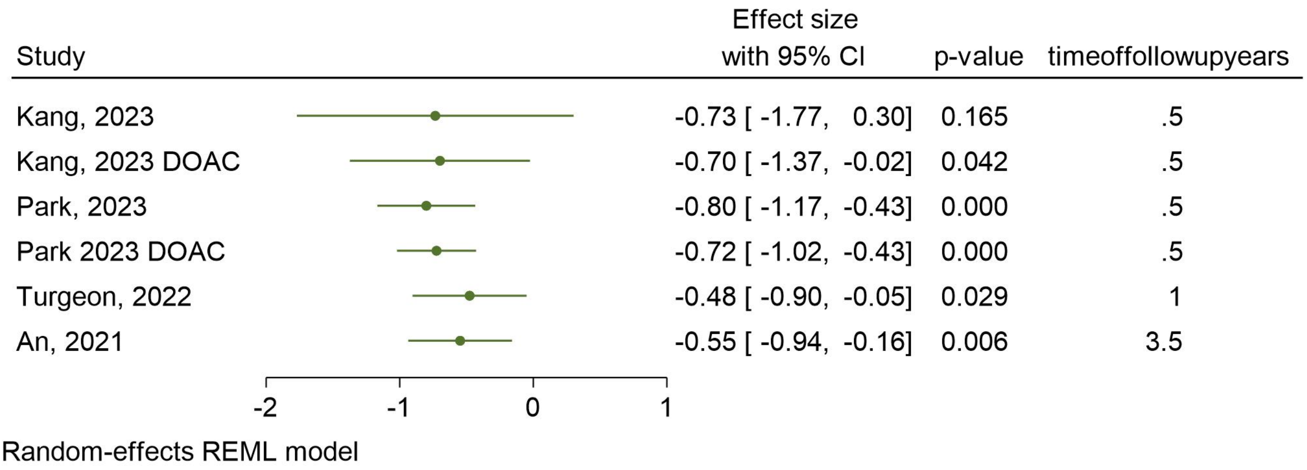

Figure S19. Meta-analyses restricted to studies accounting for reverse causation.

a. Recurrent Venous Thromboembolism

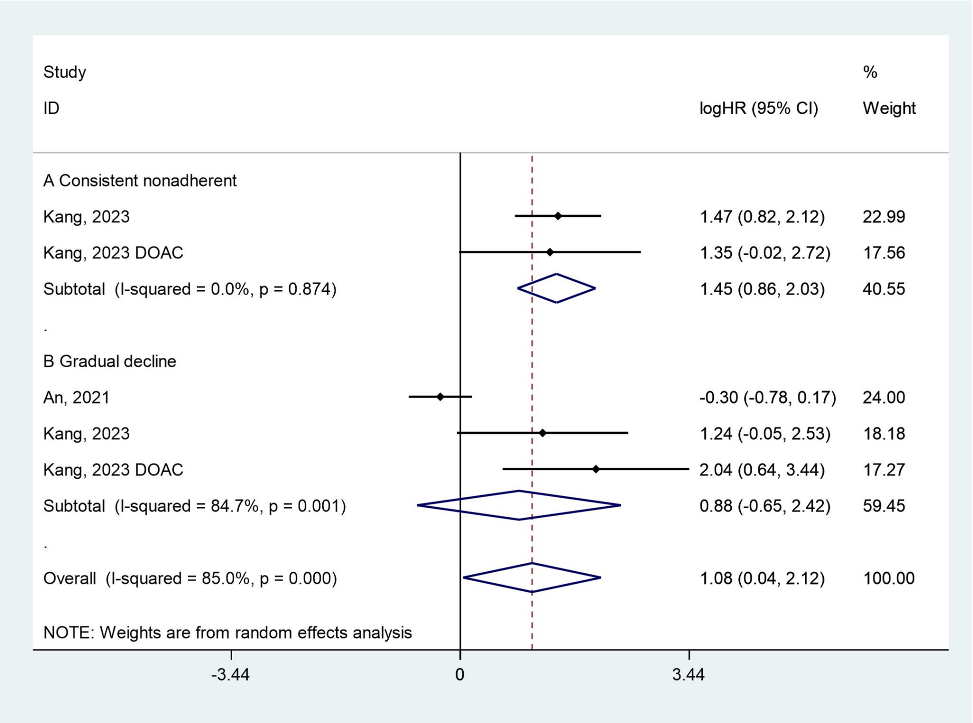

b. Major bleeding

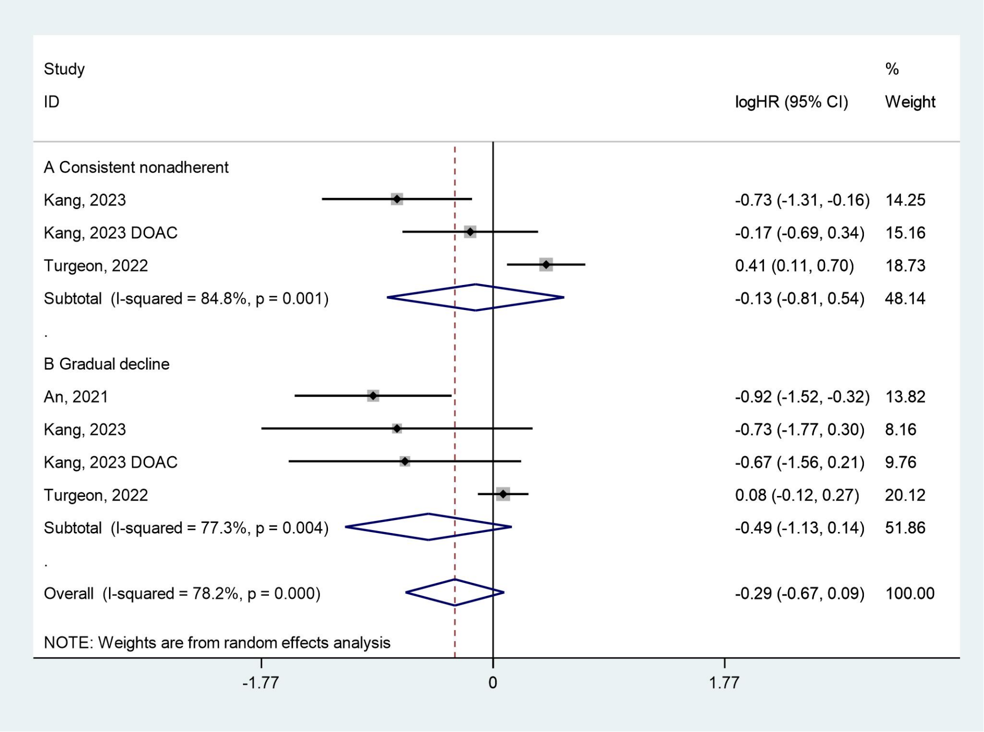

Figure S20. Meta-analyses restricted to studies with low risk of bias.

a. all-cause mortality

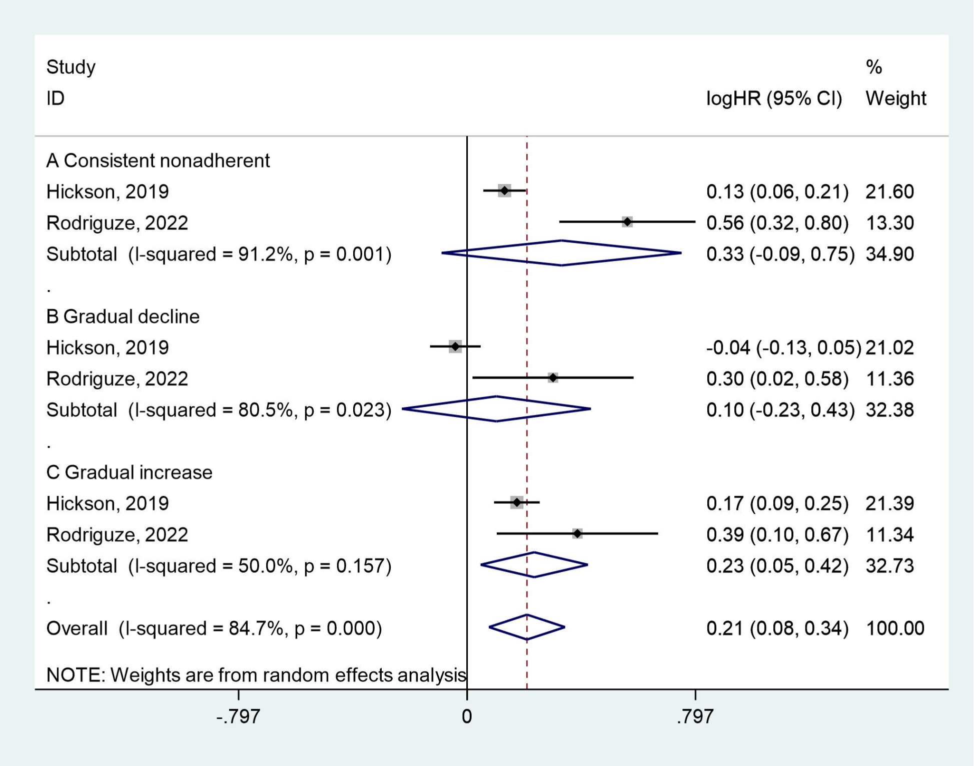
Study
ID
logHR (95% CI)
%
Weight
A Consistent nonadherent
Hickson, 2019
0.13 (0.06, 0.21)
21.60
Rodriguze, 2022
0.56 (0.32, 0.80)
13.30
Subtotal (I-squared = 91.2%, p = 0.001)
0.33 (-0.09, 0.75)
34.90
B Gradual decline
Hickson, 2019
-0.04 (-0.13, 0.05)
21.02
Rodriguze, 2022
0.30 (0.02, 0.58)
11.36
Subtotal (I-squared = 80.5%, p = 0.023)
0.10 (-0.23, 0.43)
32.38
C Gradual increase
Hickson, 2019
0.17 (0.09, 0.25)
21.39
Rodriguze, 2022
0.39 (0.10, 0.67)
11.34
Subtotal (I-squared = 50.0%, p = 0.157)
0.23 (0.05, 0.42)
32.73
Overall (I-squared = 84.7%, p = 0.000)
0.21 (0.08, 0.34)
100.00
NOTE: Weights are from random effects analysis
-.797
0
.797

Supplementary section 5. PRISMA 2020 checklist

Section and Topic	Item #	Checklist item	Location where item is reported
**TITLE**			
Title	1	Identify the report as a systematic review.	1
**ABSTRACT**			
Abstract	2	See the PRISMA 2020 for Abstracts checklist.	1
**INTRODUCTION**			
Rationale	3	Describe the rationale for the review in the context of existing knowledge.	2,3
Objectives	4	Provide an explicit statement of the objective(s) or question(s) the review addresses.	2,3
**METHODS**			
Eligibility criteria	5	Specify the inclusion and exclusion criteria for the review and how studies were grouped for the syntheses.	4
Information sources	6	Specify all databases, registers, websites, organisations, reference lists and other sources searched or consulted to identify studies. Specify the date when each source was last searched or consulted.	3,4
Search strategy	7	Present the full search strategies for all databases, registers and websites, including any filters and limits used.	4
Selection process	8	Specify the methods used to decide whether a study met the inclusion criteria of the review, including how many reviewers screened each record and each report retrieved, whether they worked independently, and if applicable, details of automation tools used in the process.	4,5
Data collection process	9	Specify the methods used to collect data from reports, including how many reviewers collected data from each report, whether they worked independently, any processes for obtaining or confirming data from study investigators, and if applicable, details of automation tools used in the process.	4,5
Data items	10a	List and define all outcomes for which data were sought. Specify whether all results that were compatible with each outcome domain in each study were sought (e.g. for all measures, time points, analyses), and if not, the methods used to decide which results to collect.	3,4

Section and Topic	Item #	Checklist item	Location where item is reported
	10b	List and define all other variables for which data were sought (e.g. participant and intervention characteristics, funding sources). Describe any assumptions made about any missing or unclear information.	3,4
Study risk of bias assessment	11	Specify the methods used to assess risk of bias in the included studies, including details of the tool(s) used, how many reviewers assessed each study and whether they worked independently, and if applicable, details of automation tools used in the process.	5
Effect measures	12	Specify for each outcome the effect measure(s) (e.g. risk ratio, mean difference) used in the synthesis or presentation of results.	5
Synthesis methods	13a	Describe the processes used to decide which studies were eligible for each synthesis (e.g. tabulating the study intervention characteristics and comparing against the planned groups for each synthesis (item #5)).	4,5
	13b	Describe any methods required to prepare the data for presentation or synthesis, such as handling of missing summary statistics, or data conversions.	5
	13c	Describe any methods used to tabulate or visually display results of individual studies and syntheses.	5
	13d	Describe any methods used to synthesize results and provide a rationale for the choice(s). If meta-analysis was performed, describe the model(s), method(s) to identify the presence and extent of statistical heterogeneity, and software package(s) used.	5,6
	13e	Describe any methods used to explore possible causes of heterogeneity among study results (e.g. subgroup analysis, meta-regression).	6
	13f	Describe any sensitivity analyses conducted to assess robustness of the synthesized results.	6
Reporting bias assessment	14	Describe any methods used to assess risk of bias due to missing results in a synthesis (arising from reporting biases).	5,6
Certainty assessment	15	Describe any methods used to assess certainty (or confidence) in the body of evidence for an outcome.	5,6
**RESULTS**			

Section and Topic	Item #	Checklist item	Location where item is reported
Study selection	16a	Describe the results of the search and selection process, from the number of records identified in the search to the number of studies included in the review, ideally using a flow diagram.	6,7, Supplementary eFigure 1
	16b	Cite studies that might appear to meet the inclusion criteria, but which were excluded, and explain why they were excluded.	6,7
Study characteristics	17	Cite each included study and present its characteristics.	7-12
Risk of bias in studies	18	Present assessments of risk of bias for each included study.	13, Supplementary p14-18
Results of individual studies	19	For all outcomes, present, for each study: (a) summary statistics for each group (where appropriate) and (b) an effect estimate and its precision (e.g. confidence/credible interval), ideally using structured tables or plots.	13-16
Results of syntheses	20a	For each synthesis, briefly summarise the characteristics and risk of bias among contributing studies.	7-13
	20b	Present results of all statistical syntheses conducted. If meta-analysis was done, present for each the summary estimate and its precision (e.g. confidence/credible interval) and measures of statistical heterogeneity. If comparing groups, describe the direction of the effect.	13-17,Supplementary p18-p37
	20c	Present results of all investigations of possible causes of heterogeneity among study results.	17
	20d	Present results of all sensitivity analyses conducted to assess the robustness of the synthesized results.	17, Supplementary p18-p37
Reporting biases	21	Present assessments of risk of bias due to missing results (arising from reporting biases) for each synthesis assessed.	17
Certainty of evidence	22	Present assessments of certainty (or confidence) in the body of evidence for each outcome assessed.	17
**DISCUSSION**			
Discussion	23a	Provide a general interpretation of the results in the context of other evidence.	17-19
	23b	Discuss any limitations of the evidence included in the review.	19-20

Section and Topic	Item #	Checklist item	Location where item is reported
	23c	Discuss any limitations of the review processes used.	19-20
	23d	Discuss implications of the results for practice, policy, and future research.	18-20
**OTHER INFORMATION**			
Registration and protocol	24a	Provide registration information for the review, including register name and registration number, or state that the review was not registered.	2
	24b	Indicate where the review protocol can be accessed, or state that a protocol was not prepared.	2,3
	24c	Describe and explain any amendments to information provided at registration or in the protocol.	3
Support	25	Describe sources of financial or non-financial support for the review, and the role of the funders or sponsors in the review.	21
Competing interests	26	Declare any competing interests of review authors.	21
Availability of data, code and other materials	27	Report which of the following are publicly available and where they can be found: template data collection forms; data extracted from included studies; data used for all analyses; analytic code; any other materials used in the review.	21
